# Gut microbiome in early life and bone health outcomes at age 6: a Danish mother-child cohort study

**DOI:** 10.1093/jbmr/zjaf108

**Published:** 2025-08-12

**Authors:** Pantalius Nji Che, Jie Jiang, Monique Breslin, Michael Thompson, Rebecca K Vinding, Jakob Stokholm, Lieke E J M Scheepers

**Affiliations:** Menzies Institute for Medical Studies, University of Tasmania, Hobart, 7000, Australia; Copenhagen Prospective Studies on Asthma in Childhood (COPSAC), University of Copenhagen, Copenhagen, 2820, Denmark; Menzies Institute for Medical Studies, University of Tasmania, Hobart, 7000, Australia; Menzies Institute for Medical Studies, University of Tasmania, Hobart, 7000, Australia; Department of Endocrinology, Royal Hobart Hospital, Hobart, 7000, TAS, Australia; Copenhagen Prospective Studies on Asthma in Childhood (COPSAC), University of Copenhagen, Copenhagen, 2820, Denmark; Department of Pediatrics and Adolescent Medicine, Copenhagen University Hospital − Herlev and Gentofte, Herlev, 2730, Denmark; Copenhagen Prospective Studies on Asthma in Childhood (COPSAC), University of Copenhagen, Copenhagen, 2820, Denmark; Menzies Institute for Medical Studies, University of Tasmania, Hobart, 7000, Australia

**Keywords:** gut microbiome, BMD, bone mineral content, childhood, longitudinal

## Abstract

The gut microbiome is associated with bone mass acquisition, yet evidence in childhood remains limited. Given that lower peak bone mass predicts osteoporosis in later life, understanding early influences is important. This analysis explores the association between the early life gut microbiome and bone health in later childhood. Data were obtained from 700 children recruited in pregnancy and followed prospectively within the Copenhagen Prospective Studies on Asthma in Childhood_2010_ cohort, a population-based mother-child cohort. The infant gut microbiome was measured at 1 wk (*n* = 445), 1 mo (*n* = 492), 1 yr (*n* = 508), 4 yr (*n* = 350), and 6 yr (*n* = 327) of age by 16S ribosomal ribonucleic acid amplicon sequencing targeting the fourth variable region. Total body less head BMD and area-adjusted BMC were measured by DXA at 6 yr of age. Associations were investigated by multiple linear regression, permutational analysis of variance, differential abundance analysis, and Random Forest machine learning. There were few associations between the early-life gut microbiome and bone health outcomes at age 6. We found negative associations between alpha (within-sample) diversity and area-adjusted BMC at 4 yr. Beta (between-sample) diversity of the gut microbiome at 6 yr was associated with concurrent BMD. *Escherichia-Shigella* abundance at 1 mo of age was associated with lower BMD. *Sutterella* abundance at 1 yr was associated with lower BMD and area-adjusted BMC at 6 yr. There were no other associations between the gut microbiome and bone outcome measures at any time point. In a well-powered unselected cohort study with longitudinal sampling of the gut microbiome, there were some suggestive but no consistent associations between the early gut microbiome and bone health outcomes at 6 yr of age.

## Graphical Abstract

**Graphical Abstract:**
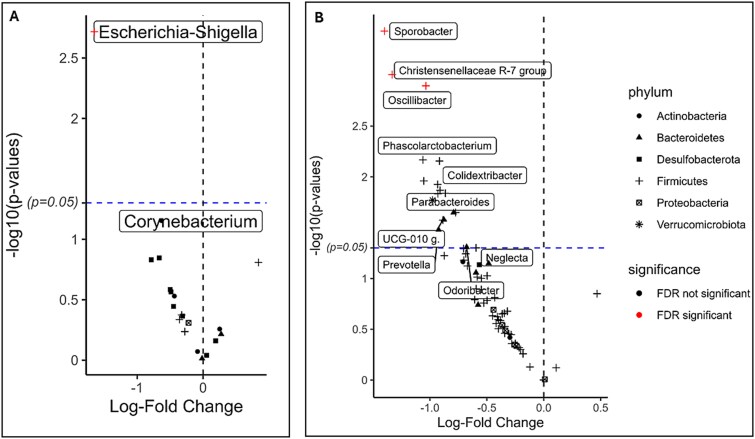

## Introduction

Emerging evidence suggests a link between the gut microbiome and bone health.^1^^,^^2^ This association is particularly important when considering osteoporosis, a condition characterized by fragile bone that affects 19.7% of the global population.^3^ Osteoporosis increases the risk of fracture, which can result in chronic pain, disability, and is associated with premature death.^3–5^ Bone development accelerates during childhood and adolescence, achieving peak bone mass (PBM) by the mid-20s.^5^^,^^6^ Peak bone mass is a key predictor of osteoporosis risk.^4^^,^^5^ Understanding the gut microbiome’s role in childhood bone acquisition is therefore important for reducing the risk of osteoporosis later in life.

Several mechanisms to explain the association between gut microbiome and bone homeostasis have been proposed.^7^ These are primarily mediated via microbial metabolites, which may exert an immunomodulatory effect or influence circulating endocrine hormone concentration. For example, microbial-produced short chain fatty acids (SCFA) such as butyrate can induce regulatory T cells (Treg) and inhibit T helper 17 cells (Th17) production.^7^ Treg inhibit osteoclastogenesis by suppressing monocytes differentiation into osteoclasts, thereby promoting bone formation, while Th17 cells promote osteoclastogenesis by producing proinflammatory cytokines (IL-17, RANKL), which increase bone loss.^8^ Short chain fatty acids also increase intestinal calcium absorption, reducing parathyroid hormone secretion and thereby facilitating bone acquisition.^7^ Additionally, the gut microbiome also influences systemic levels of insulin-like growth factor 1, which upregulates osteoblast activity.^9^

In humans, there are limited observational^10^^,^^11^ and interventional^12^^,^^13^ data examining the gut microbiome’s impact on bone health. These studies are often cross-sectional case-control studies, comparing osteoporotic cases with healthy controls primarily in adults and post-menopausal women.^14^ Such studies do not exclude the probability of reverse causality. Additionally, factors like diet may influence both the microbiome and bone health, complicating the disentanglement of the gut microbiome’s effect.

Despite being a crucial developmental stage, to our knowledge, only 2 studies have evaluated the association between gut microbiome and bone health in childhood. A small cross-sectional study on 236 healthy 6-9-yr-old city-dwelling Chinese children showed a negative association between *Blautia* and *Lachnoclostridium* and BMD.^15^ The second was a published abstract of a cross-sectional study of 2173 healthy ~9.8-yr-old city-dwelling Dutch children, which showed a positive association between *Enterococcus faecium* and BMD.^16^ In addition to being cross-sectional, these studies focused on later childhood. This highlights the need for longitudinal evidence in early childhood with a clear temporal sequence to eliminate the likelihood of reverse causality.

We hypothesize that the early-life gut microbiome has a role in shaping later bone health outcomes in childhood. Therefore, the aim of this study is to investigate the association between the gut microbiome in early life and subsequent bone health outcomes in childhood.

## Materials and methods

### Study design and population

**Figure 1:**
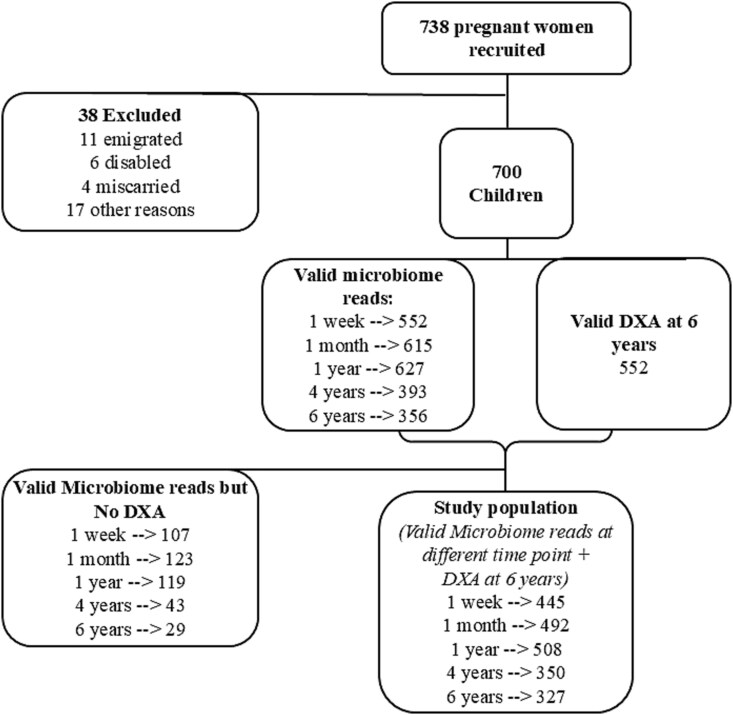
Flow chart of study participants. Participants included in study were those with valid microbiome data for at least 1 time point and valid DXA bone health measure at 6 yr of age. Of the 700 children followed up, 552 had a valid whole-body DXA scan. Of these, those with valid microbiome reads at either time points comprised the study population.

The Copenhagen Prospective Studies on Asthma in Childhood (COPSAC)_2010_ cohort is a prospective population-based mother-child cohort in Denmark. A total of 738 women and their families were recruited in pregnancy and 700 children were followed up during regular intervals at the COPSAC research unit from 2008/2011 at 1 wk, 1, 3, 6, 12, 18, 24, 30, and 36 mo, yearly thereafter to age 6 yr.^17^ All biological samples, clinical measurements, and diagnoses during clinical visits were conducted by COPSAC study physicians during scheduled visits. The present study consists of 549 children with at least 1 valid microbiome sample at age 1 wk, 1 mo, 1 yr, 4 yr, or 6 yr and a valid DXA scan (GE Healthcare, Fairfield, CT, USA) at 6 yr of age **(**Figure 1**,**  Figure S1)**.** The cohort consisted of 5 twin pairs, but only 1 child from each pair was included.

### Study outcome

The present study assesses bone health based on DXA measurements obtained at the 6-yr visit. DXA results were quality controlled as previously shown.^18^^,^^19^ Total body less head (TBLH) DXA results were considered, including BMD and aBMC, which was derived as a measure of volumetric BMD by using linear regression to adjust BMC for bone area and adding the residuals to the mean BMC.^20^ We evaluated bone health outcomes as continuous variables to explore trends and categorical variables for non-linear patterns. We explored non-linear patterns by categorizing the outcome data into low vs high (median split) and into tertiles (low, medium, and high).

### Fecal sample collection and sequencing

Fecal samples were collected at age 1 wk, 1 mo, 1 yr, 4 yr, and 6 yr, at the COPSAC research clinic or by parents at home with detailed instructions.^21^ Upon arrival, each sample was homogenized in 1 mL of 10% vol/vol glycerol (Statens Serum Institut, Copenhagen, Denmark) and preserved at −80 °C. DNA from fecal samples was extracted on an epMotion 5075 using MoBio PowerSoil Kits (MO-BIO Laboratories, Inc., Carlsberg, CA, USA) followed by gene amplicon sequencing of the 16S rRNA V4 region. Fastq-files were demultiplexed by MiSeq Controller software (Illumina Inc.), the primers and adaptors in sequencing reads were removed using Cutadapt.^22^ The determination of amplicon sequence variants (ASVs) was performed on QIIME2 Core 2020.11 platform^23^ with the Divisive Amplicon Denoising Algorithm 2 (DADA2) pipeline.^24^ Taxonomy assignments were performed as previously described.^25^

### Covariates

The following information was collected from mothers by in-person interviews during clinical visits at the research clinic and complemented by pregnancy records: Mother’s age at birth, education level at birth (low/medium/high), biannual household income (below 400.000 DKK (Danish krone), 400.000-600.000 DKK, 600.000-800.000 DKK, 800.000-1.000.000 DKK, above 1.000.000 DKK), mode of delivery (vaginal delivery/planned cesarean/emergency cesarean), gestational age (weeks), birth weight (kg), antibiotics at birth (for the mother, yes/no), antibiotics at birth (for the child, yes/no), duration of exclusive breastfeeding (months), child sex (female/male), child’s race (Caucasian/non-Caucasian), Child’s actual age at DXA scan visit (years), and log (library size). Information on antibiotic use was validated against the Danish National Prescription Registry.

Height at DXA scan visit (cm) was measured using a stadiometer (Harpenden, Holtain Ltd, Crymych, Dyfed, Wales). Total body less head bone free mass (kg) was measured by DXA. Socio-economic status was quantified by generating a PCA of maternal age at birth, maternal education level at birth, and biannual household income.

### Statistical analysis

All statistical analyses were performed in R v.4.3.0, including the “phyloseq” package v.1.46.0^26^ for microbiome analysis and ggplot2 v.3.5.1.^27^ for visualization. Within-sample (alpha) diversity was assessed using observed richness (accounts for number of unique taxa only) and Faith’s Phylogenetic Diversity (PD), which accounts for both the number of unique taxa and their evolutionary relationships. The association between alpha diversity at each time point and continuous bone health outcomes was evaluated by linear regression (Figure S1). Difference in alpha diversity (PD) by bone health categories was evaluated by ANOVA, and Tukey’s Honest Significant Difference (TukeyHSD) post hoc test was used to compare group pairs. Compositional differences between samples (beta diversity) were assessed using the weighted UniFrac metric, which accounts for both the relative abundance of taxa and their evolutionary relationships. Abundances for beta diversity calculations were log-transformed (after adding pseudo-count of +1) to mitigate the influence of dominant taxa. To visualize the association between the beta diversity and categorical bone health outcomes, we used principal coordinates analysis plots. To test associations, we used permutational multivariate analysis of variance (PERMANOVA) with 999 permutations using “adonis2” from the vegan package v.2.6-4.^28^^,^^29^

We furthermore analyzed individual bacterial genera in relation to bone health outcomes. At genus level, we filtered the taxa based on the threshold Prevalence ≥25%*(1e-4/Mean Relative Abundance)^0.5^36^ and centered-log-ratio transformed the abundances for downstream analysis (by adding 1 to every abundance value, dividing by the geometric mean of all values in the sample, and taking log of the ratio). To test the association between individual genera and continuous bone health outcomes, we used linear regression. To test non-linear patterns, we used categorical outcomes with the differential abundance (DA) test (R package “DAtest”)^43^ across all timepoints. DAtest systematically benchmarks differential analysis methods by comparing their performance in terms of power, false positive rate, and area under the curve (AUC), which enabled us to select the most appropriate method for their specific dataset. We chose LIMMA-ALR (linear models for microarray and omics data-additive log ratio) based on optimal performance scores.

We used 10-fold cross-validated Random Forest machine learning (R package “randomForest” v4.7-1.1^30^) to identify combination of genera that predict later bone health. Data were divided into 10 sets, the model was trained on 9, tested on 1, and repeated 10 times. Model performance was evaluated based on R-squared, root mean squared error (RMSE) and mean absolute error values. Following cross-validation, model parameters (number of trees and “mtry”) that minimized the RMSE were used for analysis. Feature importance was used to identify the microbial taxa most predictive of bone health.

All continuous variables were summarized as means ± standard deviation if normally distributed or median and interquartile range if not normally distributed. Categorical variables were summarized as percentages. Missing data for covariates were treated as missing observations. Adjustments for multiple testing were done by Benjamini-Hochberg correction with False Discovery Rate (FDR) cut-off of 0.05.

We identified potential confounders and mediators a priori based on the graphical criteria for confounding by visualizing a directed acyclic graph (Figure S2). Analyses were adjusted for child’s sex, race (Caucasian/non-Caucasian), and age and height at the DXA scan visit. We additionally adjusted for socio-economic status by including the first component of a principal component analysis of child’s family income, and mother’s age and educational level at time of delivery. To account for varying sequencing depth, we also adjusted for log (library size).

### Sensitivity analyses

Sensitivity analyses were performed to assess the robustness of findings to methodological choices: (1) Observed richness was used as alternative measure for within-sample diversity, (2) unweighted UniFrac was employed as an alternative measure for between-sample diversity, and (3) non-linear patterns were additionally investigated by categorizing the outcome variable into low vs high (median split) (Figure S3a and b).

### Exploratory analysis

To assess the robustness of observed associations and account for the potential mediation effect by body composition, we adjusted for bone-free mass (BFM)—defined as the sum of lean and fat mass—as an additional covariate in the models assessing bone outcomes.

Recognizing the compositional nature of microbiome data and its implications for interpretation, we additionally examined whether the observed associations were primarily driven either by taxon abundance or by the presence or absence of the taxa. To do this, we transformed the relative abundance data into binary indicators (presence/absence) for key taxa previously identified in primary analyses. We assessed the effect of taxon presence/absence using linear mixed-effects models incorporating a random intercept for participant ID to account for repeated measures across time. This was followed by multiple linear regression restricted to the specific time point(s) at which the taxon’s abundance was significantly associated with bone outcomes. Finally, to isolate the effect of microbial abundance in the absence of variability introduced by non-carriers, we re-estimated the associations in the subset of participants with detectable levels of the taxon and compared the estimates to those from the full sample at the corresponding time point.

## Results

### Baseline characteristics of study participants

Baseline characteristics of the study population are summarized in Table S1. At enrolment, the mean maternal age was 32.4 yr (SD, 4.3), with 160 mothers (29.1%) holding a university degree. The average gestational age at birth was 279.2 d (SD, 12.0). Of the children included in the study, 270 (49.2%) were boys. At 6 yr of age, the mean TBLH BMD was 0.56 mg/cm^2^ (SD, 0.05), and the mean area-adjusted BMC (aBMC) was 543.9 g (SD, 36.6).

### Microbial abundance and prevalence in the first 6 yr of life

The most abundant and prevalent genera in decreasing order by age included: *Bacteroides, Bifidobacterium*, *Escherichia-Shigella, Klebsiella,* and *Staphylococcus* at 1 wk, *Escherichia-Shigella, Klebsiella, Veillonella, Streptococcus,* and *Clostridium* at 1 mo, *Escherichia-Shigella, Faecalibacterium, Bifidobacterium, Prevotella,* and *Akkermansia* at 1 yr, *Bacteroides, Blautia*, *Bifidobacterium, Clostridium,* and *Dialister* at 4 yr, and *Alistipes, Bifidobacterium, Dialister, Escherichia-Shigella,* and *Prevotella,* at 6 yr, respectively. The rank of different microbial genera by both abundance and prevalence according to child’s age are shown in Figure S4a−e.

### Microbial diversity and bone health outcomes

As shown in Table 1, we observed that higher alpha diversity (PD) at 4 yr of age was associated with lower aBMC. Using observed richness alpha diversity index, we equally reported negative associations at 4 yr for BMD and aBMC (Table S2). At 1-wk, 1-mo, and 6-yr of age, alpha diversity was not associated with bone health outcomes.

**Table 1:** Alpha diversity and beta diversity association with bone measures, model excluding bone free mass.

**Diversity index**		**Measure of bone health at age 6**	**1 wk** ***n* = 445**	**1 mo** ***n* = 492**	**1 yr** ***n* = 508**	**4 yr** ***n* = 350**	**6 yr** ***n* = 327**
**Multiple linear regression; β, (95% CI) *p*-value, partial *R*** ^**2**^							
**Alpha diversity**	Faith’s phylogenetic diversity (PD)	BMD	−1.153 × 10^−3^, (−3.129 × 10^−3^, 8.241 × 10^−4)^ 0.25, 0.3%	5.288 × 10^−4^, (−1.657 × 10^−3^, 2.714 × 10^−3^) 0.63, 0.05%	−1.251 × 10^−3^, (−3.11 × 10^−3^, 6.107 × 10^−4^) 0.19, 0.3%	−1.003 × 10^−3^, (−2.476 × 10^−3^, 4.708 × 10^−4^) 0.18, 0.5%	−7.550 × 10^−4^, (−2.527 × 10^−3^, 1.017 × 10^−3^) 0.40, 0.2%
		aBMC	−1.10, (−3.06, 0.86) 0.27, 0.3%	0.46, (−1.71, 2.63) 0.68, 0.04%	−0.96, (−2.80, 0.89) 0.310, 0.2%	−1.47, (−3.03, −0.10) **0.037**^a^, 1.3%	−0.82, (−2.59, 0.95), 0.36, 0.3%
**PERMANOVA (for all participants); *F*-score, partial *R*^2^, *p*-value**							
**Beta diversity**	Weighted UniFrac	BMD	1.76, 0.4%, 0.08	0.88, 0.2%, 0.53	0.89, 0.2%, 0.53	1.64, 0.5%, 0.07	1.94, 0.6%, **0.027**^a^
		aBMC	1.12, 0.3%, 0.31	0.63, 0.1%, 0.80	1.01, 0.2%, 0.40	0.89, 0.3%, 0.51	1.23, 0.4%, 0.22

Abbreviations: aBMC, area-adjusted BMC. Associations between alpha diversity and bone health outcomes were assessed using linear regression, while associations for beta diversity were evaluated using permutational analysis of variance (PERMANOVA). Models were adjusted for child’s sex, race, socio-economic status of the family, and age, and height at the time of the DXA scan, and log (library size). Partial *R*^2^ values indicate the proportion of variance in the bone health outcome uniquely explained by the diversity measure after accounting for other covariates in the model. Summary statistics are presented for each time point. Bold values indicate results that are statistically significant, with nominally significant results denoted by.

a for *p* < .05.

**Figure 2:**
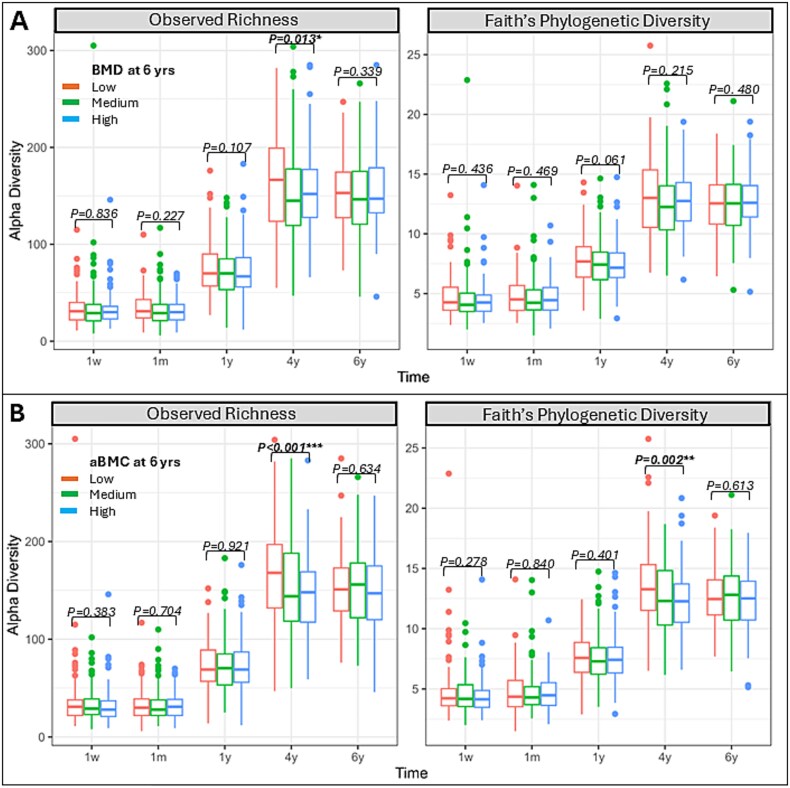
Alpha diversity measures vs bone health outcomes (tertiles) by child’s age. X-axis shows the different time points of the gut microbiome measurement and the y-axis shows the microbial diversity (Faith’s phylogenetic diversity index (PD) and observed richness for comparison). Each box represents a tertile group of bone health outcome measure (BMD or aBMC). Red = low, green = medium, blue = high bone health outcome measure. ANOVA was used for test of significance between groups and TukeyHSD for post-hoc pair-wise group comparison. Adjustment was done for child’s sex, race, socio-economic status, and age, height, and bone free mass at DXA visit, and log (library size). Observed richness and PD are measures of alpha (within-sample) diversity with PD additionally capturing phylogenetic relatedness of genera (A) alpha diversity by BMD tertiles. (B) Apha diversity by aBMC tertiles. Nominally significant associations are indicated by ** for *p* < .01, *** for *p* < .001. Abberiavtions: aBMC; area-adjusted BMC.

Grouping according to bone health tertiles, we observed that children in the low bone tertile had a higher alpha diversity at age 4 compared to those in the medium tertile (Figure 2), except for the association between PD with BMD (*p* = .215). At all other time points (1-wk, 1-mo, 1 yr and 6 yr), we did not observe an association between alpha diversity and measures of bone health.

For beta diversity, significant differences in gut microbiome composition were observed at age 6 among children with varying BMD at the same age (*F* = 1.92, *R*^2^ = 0.6%, *p* = .026) (Table 1), but we did not observe compositional differences in earlier time points associated with BMD or aBMC.

### Association of microbial abundance with continuous bone health outcomes

**Figure 3:**
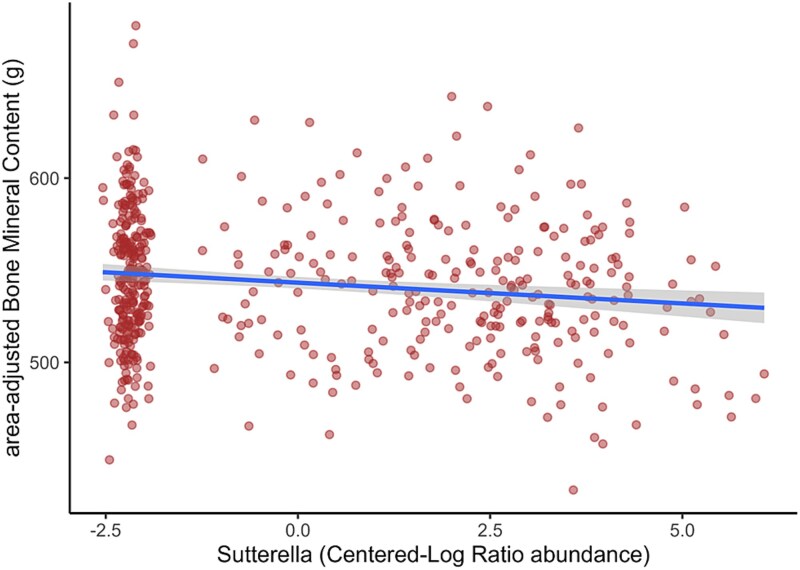
Negative association between *Sutterella* abundance at 1 yr and aBMC. X-axis represents the centered-log ratio (CLR) transformed abundance and y-axis represents total body less head area-adjusted BMC (aBMC) in grams. The shaded areas represent the 95% CIs for predictions from a linear model. Adjustment was done for child’s sex, race, socio-economic status, and age, height, bone free mass at DXA visit, and log (library size). Adjusted for multiple testing by Benjamini-Hochberg.

Based on our microbial prevalence-abundance cut-off, we selected 17, 21, 52, 67, and 72 most prevalent and abundant genera at 1 wk, 1 mo, 1 yr, 4 yr, and 6 yr, respectively, for further analysis (Figure S4a−e). We examined each genus at each time point for linear associations with BMD and aBMC at age 6 to identify potential predictors of bone health (Table 2, Figure S5a−c). While many taxa were found nominally significant, only *Sutterella* at 1 yr was associated with lower BMD (β: −2.329 × 10^−3^; 95% CI, −3.616 × 10^−3^ to −1.043 × 10^−3^, after correction for multiple testing: *p*.adj = .021) and aBMC (β: −2.46; 95% CI, −3.73 to −1.19, after correction for multiple testing: *p*.adj = .008) and it independently contributed to 2.5% and 2.8% of the variance in BMD and aBMC, respectively (Table 2, Figure 3).

### Association of microbial abundance with bone health outcome (categorical analyses)

**Figure 4:**
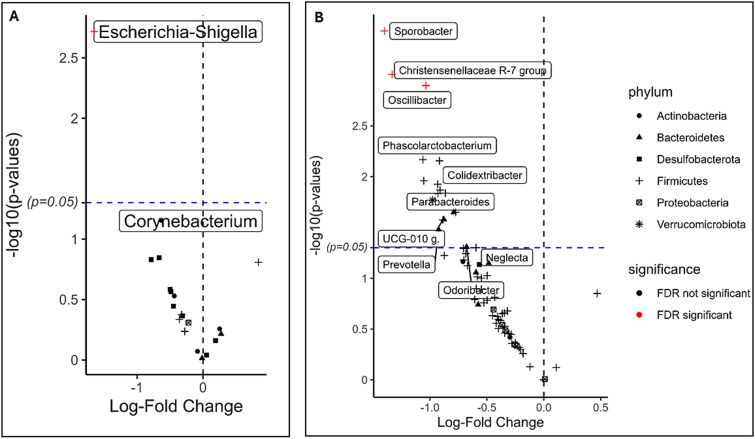
Volcano plots showing differentially abundant genera with tertiles of bone health outcomes. The x-axis shows the change in mean abundance of the genera between groups, and the y-axis shows the -log10 (pval) (higher is more significant). Bone health outcomes are grouped into tertiles, dividing the data into 3 approximately equal groups – low, medium, and high bone health measures. The DA analysis between bone groups was done by linear models for micro-array and omics analysis with additive log ratio transformation (LIMMA-ALR). Analyses were adjusted for child’s sex, socio-economic status of child’s family, child’s height, total body less head bone-free mass, child’s race, and child’s age at DXA scan, log (library size). The dashed vertical line represents zero change in mean abundance between bone groups. The dashed horizontal line represents the nominal *p*-value cut off, showing that some genera were nominally significant (black dots above line; *p* < .05) and others remained significant after FDR correction (red dots; *p*.adjust<0.05). (A) Comparing 1-mo genera in low (n = 141) vs high (n = 145) tertiles of BMD. (B) Comparing 4-yr genera in low (n = 112) vs medium (n = 142) tertiles of aBMC.

To assess whether specific microbial taxa were disproportionately represented in the extremes of childhood bone health, we further split the bone health outcomes into tertiles. We found several genera that were nominally differentially abundant between tertiles at various time points. One genus at 1 mo and 3 genera at 4 yr survived FDR correction (Table S3). At 1 mo, *Escherichia-Shigella* was differentially abundant in low compared to high BMD (*p*.adj = .038) (Figure 4A). At 4 yr, *Sporobacter* (*p*.adj = .024), *Christensenellaceae R-7 group* (*p*.adj = .028), and *Oscillibacter* (*p*.adj = .028) were differentially abundant in low compared to medium (Figure 4B) but not high aBMC. Whereas for BMD, no differentially abundant genera were identified among different tertiles. At 1 wk, 1 yr and 6 yr, there were no associations with either BMD or aBMC.

**Table 2:** Summary results for linear regression analysis (genera vs continuous bone health outcomes), model excluding bone free mass.

**Bacteria genera with low *p*-values (*n* = 230)**	**BMD / gcm** ^**−2**^		**aBMC / g**	
	**β [95% CI], *p*-value, partial *R*** ^**2**^	**Adjusted *p*-value**	**β [95% CI], *p*-value, partial *R*** ^**2**^	**Adjusted *p*-value**
**1 wk (*n* = 17)**	0/17 with *p* < .05	0/17 with *p*.adj < .05	0/17 with *p* < .05	0/17 with *p*.adj < .05
**NA**	NA	NA	NA	NA
**1 mo (*n* = 21)**	2/21 with *p* < .05	0/21 with *p*.adj < .05	1/21 with *p* < .05	0/21 with *p*.adj < .05
** *Escherichia-Shigella***	−1.331 × 10^−3^ [−2.406 × 10^−3^, −2.563 × 10^−4^], **.015**^a^, 1.2%	.188	−1.32 [−2.38, −0.25], **.016**^a^, 1.2%	.232
** *Klebsiella***	9.627 × 10^−4^ [1.729 × 10^−5^, 1.908 × 10^−3^], **.046**^a^, 0.8%	.322	0.83 [−0.11, 1.77], .084, 0.6%	.443
** *Streptococcus***	−8.050 × 10^−4^ [−2.340 × 10^−3^, 7.296 × 10^−4^], .303, 0.2%	.707	−0.55 [−2.08, 0.97], .475, 0.1%	.885
** *Staphylococcus***	−1.227 × 10^−3^ [−2.717 × 10^−3^, 2.626 × 10^−4^], .106, 0.5%	.446	−1.53 [−3.01, −0.06], **.042**^a^, 0.9%	.294
** *Corynebacterium***	−3.048 × 10^−3^ [−5.569 × 10^−3^, −5.260 × 10^−4^], **.018**^a^, 1.1%	.188	−2.93 [−5.43, −0.42], **.022**^a^, 1.1%	.232
**1 yr (*n = 52*)**	5/52 with *p* < .05	0/52 with *p.*adj < .05	3/52 with *p <* .05	1/52 with *p.*adj < .05
** *Sutterella***	−2.329 × 10^−3^ [−3.616 × 10^−3^, −1.043 × 10^−3^], **.0004**^c^, 2.5%	**.021** ^a^	−2.46 [−3.73, −1.19], **.0002**^c^, 2.8%	**.008** ^b^
** *[Eubacterium] hallii group***	2.010 × 10^−3^ [6.344 × 10^−4^, 3.385 × 10^−3^], **.004**^b^, 1.6%	.111	1.89 [0.53, 3.25], **.007**^b^, 1.5%	.175
** *Monoglobus***	2.426 × 10^−3^ [6.777 × 10^−4^, 4.175 × 10^−3^], **.007**^b^, 1.5%	.115	2.10 [0.36, 3.84], **.038**^a^, 0.9%	.432
** *Bifidobacterium***	1.343 × 10^−3^ [−3.203 × 10^−4^, 3.006 × 10^−3^], .113, 0.5%	.541	1.15 [−0.50, 2.79], .173, 0.4%	.895
** *Parasutterella***	1.567 × 10^−3^ [2.650 × 10^−4^, 2.869 × 10^−3^], **.018**^a^, 1.1%	.192	1.37 [0.08, 2.66], **.038**^a^, 0.9%	.432
** *Oscillibacter***	−1.680 × 10^−3^ [−3.349 × 10^−3^, −1.090 × 10^−5^], **.049**^a^, 0.8%	.421	−1.03 [−2.69, 0.62], .221, 0.3%	.895
** *Flavonifractor***	−1.536 × 10^−3^ [−3.444 × 10^−3^, 3.723 × 10^−4^], .114, 0.5%	.114	−1.00 [−2.89, 0.89], .300, 0.2%	.895
** *Ruminococcaceae g.***	−2.241 × 10^−3^ [−3.999 × 10^−3^, −4.826 × 10^−4^], **.013**^a^, 1.2%	.164	−1.82 [−3.56, −0.07], **.042**^a^, 0.8%	.432
** *Butyricicoccus***	1.582 × 10^−3^ [−1.696 × 10^−4^, 3.333 × 10^−3^], .077, 0.6%	.487	1.53 [−0.21, 3.26], .084, 0.6%	.729
**4 yr (*n = 67*)**	6/67 with *p <* .05	0/67 with *p.*adj < .05	8/67 with *p <* .05	0/67 with *p.*adj < .05
** *Oscillibacter***	−2.307 × 10^−3^ [−4.622 × 10^−3^, 8.392 × 10^−6^], .051, 1.1%	.593	−2.86 [−5.17, −0.56], **.015**^a^, 1.7%	.253
** *Family* × *III AD3011 group***	−2.105 × 10^−3^ [−4.138 × 10^−3^, −7.101 × 10^−5^], **.043**^a^, 1.2%	.593	−2.54 [−4.56, −0.51], **.014**^a^, 1.7%	.253
** *Colidextribacter***	−1.676 × 10^−3^ [−3.848 × 10^−3^, 4.965 × 10^−4^], .130, 0.7%	.706	−2.23 [−4.39, −0.06], **.044**^a^, 1.2%	.445
** *Clostridia g.***	−1.536 × 10^−3^ [−4.119 × 10^−3^, 1.047 × 10^−3^], .243, 0.4%	.864	−2.23 [−4.81, 0.34[, .089, 0.8%	.580
** *Odoribacter***	−2.620 × 10^−3^ [−4.524 × 10^−3^, −7.152 × 10^−4^], **.007**^b^, 2.1%	.480	−2.48 [−4.38, −0.57], **.011**^a^, 1.9%	.253
** *Lachnobacterium***	−2.306 × 10^−3^ [−4.144 × 10^−3^, −4.562 × 10^−4^], .015^a^, 1.7%	.492	−2.58 [−4.42, −0.73], **.006**^b^, 2.2%	.253
** *Incertae Sedis***	−1.563 × 10^−3^ [−3.626 × 10^−3^, 4.999 × 10^−4^], .137, 0.6%	.706	−2.07 [−4.13, −0.01], **.048**^a^, 1.1%	.445
** *Pseudoflavonifractor***	−1.862 × 10^−3^ [−3.759 × 10^−3^, 3.443 × 10^−5^], .054, 1.1%	.593	−2.05 [−3.94, −0.16[, **.035**^a^, 1.3%	.445
** *Lachnospiraceae g.***	−2.979 × 10^−3^ [−6.107 × 10^−3^, 1.495 × 10^−4^], .062, 1.0%	.593	−2.86 [−5.99, 0.26], .073, 0.9%	.540
** *Bacteroides***	−1.010 × 10^−3^ [−3.098 × 10^−3^, 1.078 × 10^−3^], .342, 0.3%	.955	−0.66 [−2.75, 1.43], .536, 0.1%	.842
**6 yr (*n = 72*)**	3/72 with *p <* .05	0/72 with *p.*adj < .05	1/72 with *p <* .05	0/72 with *p.*adj < .05
** *Terrisporobacter***	3.073 × 10^−3^ [8.922 × 10^−4^, 5.253 × 10^−3^], **.006**^b^, 2.3%	.424	1.93 [−0.26, 4.12], .084, 0.9%	.917
** *[Eubacterium] eligens group***	1.384 × 10^−3^ [−6.252 × 10^−4^, 3.393 × 10^−3^], .176, 0.6%	.805	1.56 [−0.44, 3.56], .127, 0.7%	.917
** *Prevotella***	−7.065 × 10^−4^ [−2.053 × 10^−3^, 6.403 × 10^−4^], .303, 0.3%	.956	−1.38 [−2.72, −0.04], **.043**^a^, 1.3%	.917
** *Pseudoflavonifractor***	−2.041 × 10^−3^ [−4.458 × 10^−3^, 3.748 × 10^−4^], .097, 0.9%	.805	−1.40 [−3.82, 1.01], .255, 0.4%	.917
** *Streptococcus***	1.297 × 10^−3^ [−7.623 × 10^−4^, 3.356 × 10^−3^], .216, 0.5%	.865	1.06 [−1.00, 3.11], .313, 0.3%	.917
** *Oscillospiraceae g.***	−2.592 × 10^−3^ [−4.969 × 10^−3^, −2.157 × 10^−4^], **.033**^a^, 1.4%	.805	−2.17 [−4.55, 0.20], .073, 1.0%	.917

Regression estimates show the change in bone outcome per centered-log ratio unit increase in each genus abundance. Only genera with *p* < .05 (without multiple testing adjustment) for either bone outcome measures are shown. For every genus with significant association with either aBMC or BMD, the corresponding results for the other bone outcome is shown. The covariates adjusted for included child’s sex, race, socio-economic status, and age, and height at DXA visit. Bold values indicate results that are statistically significant. Nominal significant results are indicated by

a for *p* < .05.

b for *p* < .01.

c for *p* < .001.

*p.*adj shows *p*-values adjusted for multiple testing by Benjamini-Hochberg (FDR threshold of 0.05).

### Machine learning analysis to identify jointly contributing taxa

We used Random Forest on the subsets of abundant and prevalent bacteria to explore subtle patterns in the microbiome that predict later bone health measures undetected by other methods. For aBMC, only the microbiome composition data at 1 mo of age significantly predicted aBMC at 6 yr of age (*R*^2^ = 3.3%, RMSE = 35.0, *p* = .048) (Figures S6a and b, S7a−e). The top 3 predictive genera at 1 mo were *Haemophilus, Escherichia-Shigella,* and *Klebsiella,* respectively. The test set performance for predicting aBMC at other time points, explained between 0.2% and 1.3% of aBMC, with RMSE ranging from 34.7 to 40.1 and *p*-value from .315 to .687. For BMD, there were no significant predictions by the microbiome at any timepoint with test set performance for predicting BMD explaining between 0.005% and 2.6% of variance, with RMSE ranging from 0.046 to 0.049 and *p*-value from .152 to .936. Overall, model performance at 1 yr was poor (BMD: *R*^2^ = 0.005%, RMSE = 0.047, *p* = .152; aBMC: *R*^2^ = 0.2%, RMSE = 15.2, *p* = .652). Regardless, *Sutterella*, *Eubacterium hallii group, and Monoglobus,* which were identified by other methods at the 1-year timepoint, were equally ranked top 3 predictors of 6-yr aBMC.

### Sensitivity analyses

The robustness of our findings was assessed through several sensitivity analyses. Using observed richness as alternative measures for within-sample diversity did not significantly alter the results (Table S2). Similarly, employing unweighted UniFrac as an alternative measure for between-sample diversity yielded consistent findings (Table S2). Additionally, investigating non-linear patterns by categorizing the outcome variable into low vs high (median split) confirmed the robustness of our primary results (Figure S3a and b; Figure 2).

### Exploratory analysis

Following an additional adjustment for BFM on all primary models, we observed no changes in effect directions across all microbial characteristics (Table S2; Table S4). However, for *Sutterella* at age 1 yr previously associated with both BMD and aBMC, we observed attenuation in effect sizes: for BMD, from −2.33 × 10^−3^ g/cm^2^ to −1.89 × 10^−3^ g/cm^2^ (a 19% reduction; *p* = .063), and for aBMC, from −2.46 g to −2.16 g (a 12% reduction; *p* = .025) (Table S4).

In addition, we tested the effect of *Sutterella* presence/absence and found no overall association across timepoints BMD: (Estimate = −1.301x10^−3^, *p* = .42); aBMC: (Estimate = −2.65, *p* = .10). Assessing the effect of *Sutterella* presence/absence at 1 yr showed an associated with 7.826 × 10^−3^ g/cm^2^ lower BMD (*β* = −7.826 × 10^−3^, *p* = .017) and 9.00 g lower aBMC (*β* = −9.00, *p* = .006) at 6 yr (Figure S8). In addition, when testing the association among participants with prevalent *Sutterella*, further analysis using relative abundance data showed that the associations persisted, with even larger effect sizes (BMD: *β* = −.555, *p* = .006; aBMC: *β* = −683.46, *p* = .0007) (Figure S9).

## Discussion

In this large prospective mother-child cohort, we found no consistent associations between the early gut microbiome and bone health outcomes at 6 yr. However, we observed that a higher microbiome alpha diversity at 1 and 4 yr of age was associated with lower bone health outcomes. Additionally, beta diversity at 6 yr was associated with concurrent BMD. We also identified associations between the genera *Escherichia-Shigella* at 1 mo and *Sutterella* at 1 yr with later bone health outcomes at 6 yr. While our findings suggest that the gut microbiome at in early life may have a modest contribution to bone health in later childhood, only a small proportion of its variation was explained.

This study is among the earliest to investigate the association between gut microbiome diversity during early childhood and later bone health outcomes in childhood. We found that alpha diversity—a measure of within-sample diversity—at 1 and 4 yr of age was negatively associated with bone health outcomes at 6 yr. Whereas no associations were observed at other time points. This finding contrasts with the only prior study by Chen et al.,^15^ which reported no association between alpha diversity and bone health parameters in a small cross-sectional analysis of 236 healthy Chinese children aged 6-9 yr.

In contrast to alpha diversity, we did not observe longitudinal associations between microbial community composition and bone health outcomes in the beta diversity analyses—a measure of between-sample diversity. However, we identified a cross-sectional association at 6 yr between BMD and the weighted UniFrac metric, which incorporates both microbial abundance and phylogenetic relatedness. The absence of associations using the unweighted UniFrac metric, which captures only presence/absence of taxa, suggests that differences in microbial abundance, rather than taxa prevalence, may play a concurrent role in bone health. This finding aligns with previous research on other health outcomes, including a study on childhood adiposity^31^ and an adult study on bone health,^32^ both of which reported no associations with beta diversity.

While higher microbial diversity is generally considered beneficial for host health, including bone health,^33^ results have been inconsistent. For instance, higher alpha diversity has been linked to osteopenia and osteoporosis in adults.^34^ This discrepancy may arise because the impact of the gut microbiome on bone health is not solely determined by diversity metrics. Instead, it might depend on the prevalence, abundance, and function of specific microbial taxa, which diversity metrics alone do not capture.

In the current study, we therefore examined the association between individual genera with highest relative abundance and prevalence at several time points and subsequent bone health outcomes at 6 yr of age. *Escherichia-Shigella* was among the top 5 abundant and prevalent genera at 1 wk, 1 mo, 1 yr, and 6 yr. At 1 mo of age only, the relative abundance of *Escherichia-Shigella* was associated with lower BMD and aBMC, but did not remain significant after correction for multiple testing. However, when comparing children in the lowest with highest BMD tertile, the association remained significant after FDR correction. This is in line with the cross-sectional study in Chinese children, which reported that a lower relative abundance of *Escherichia-Shigella* was associated with higher BMD of the lumbar spine, explaining 3.4% of variance.^15^ As this is the only study in children, we also consider evidence from adult studies, usually cross-sectional studies, comparing osteoporotic cases with controls, with all the associated limitations. For example, studies in postmenopausal women have consistently showed a higher relative abundance of *Escherichia-Shigella* in osteoporotic cases.^32^^,^^35^ Similarly, a study of 4054 adults aged 40-69 yr from the UK Biobank showed that *Escherichia-Shigella* was more abundant in people with lower lumbar spine BMD.^35^

Several potential biological explanations can be formulated as to why genus *Escherichia-Shigella* play a role in bone development. For example, *Escherichia-Shigella* include pathogenic bacteria that promote gastro-intestinal tract inflammation associated with infant diarrhea and dysentery, which could then be hypothesized to lead to a degree of malabsorption.^35^^,^^36^ Additionally, inflammatory cytokines that are activated as a result, eg, IL-1, IL-6, and IL − 17 are associated with increased osteoclast proliferation.^8^ This genus also produces Trimethylamine-N-oxide (TMAO) in the gut from dietary nutrients such as L-carnitine and choline, which in turn has been linked to poor bone health.^11^ Since *Escherichia-Shigella* was associated with BMD only at 1 mo and explained just ~2.0% of variation in the outcome, its clinical relevance remains uncertain and requires further replication.

We also observed that *Sutterella* at age 1 yr was negatively associated with later BMD and aBMC in both the main analyses, which used relative abundance as a continuous predictor including all participants (those with and without *Sutterella*), and in exploratory analyses that focused on abundance among those colonized with *Sutterella*. These findings suggest that while the presence of *Sutterella* may be relevant, it is primarily the relative abundance that drives the observed associations with bone outcomes. On the contrary, a small cross-sectional study on 38 postmenopausal Japanese women showed that *Sutterella* was positively associated with high serum vitamin K2, a vital component for maintaining bone quality.^37^ To our knowledge, there is no evidence on the role of *Sutterella* on general childhood health or bone health. Nonetheless, *Sutterella* abundance has been linked to gastrointestinal inflammation in 2 recent studies, which has been suggested to impair bone mineralization through osteoclast activation and bone resorption.^37^^,^^38^ On the contrary, other studies report that *Sutterella* was differentially enriched in individuals with high interleukin 13 (IL-13), a Th2 cytokine typically involved in anti-inflammatory responses. However, persistent overexpression of IL-13 has been associated with chronic inflammation and tissue remodeling in diseases such as asthma and fibrosis.^39^ Chronic IL-13-driven inflammation may disrupt bone remodeling homeostasis by enhancing osteoclastogenesis and inhibiting osteoblast differentiation, potentially contributing to reduced bone health.^40^

Several other genera were associated, but none survived FDR. At 4 yr, *Odoribacter* and *Oscillibacter*, and at 6 yr, *Prevotella*, were relatively more abundant in children with lower bone health measures, explaining a variance of 2.0%, 2.1%, and 2.0%, respectively for aBMC. However, the evidence on their role in bone health remains inconclusive. While our study found a negative association of these genera with bone measures, the literature presents conflicting findings, reporting no,^41^ positive,^32^^,^^36^ or negative associations.^10^ For instance, *Odoribacter* is recognized for its critical role in maintaining gut health,^32^ which supports nutrient absorption and metabolic functions essential for skeletal health. Similarly, studies using animal models^36^ and case-control studies in postmenopausal women have linked higher relative abundances of *Odoribacter* and *Oscillibacter* to higher BMD.^7^^,^^32^^,^^42^ Moreover, *Prevotella* has been identified in recent case-control studies as a therapeutic target for osteopenia and osteoporosis due to its SCFA production, which enhances calcium absorption.^32^

Conversely, other research has linked *Odoribacter*,^10^^,^^43^  *Oscillibacter*,^42^ and *Prevotella*^44^ with microbial dysbiosis, initiation of T-cell mediated pro-inflammatory responses, and lower BMD, particularly in peri- or postmenopausal women. These discrepancies highlight the complex, context-dependent relationships between microbial taxa and bone health. The lack of consistent associations across time points in our study suggests that these findings may be chance observations or reflect transient influences during specific stages of development. Investigating how taxa interact within microbial networks could provide deeper insights into their collective impact on bone health.

In our exploratory analysis, adjustment for BFM led to a modest attenuation of the associations between *Sutterella* and both BMD and aBMC, indicating partial mediation. This suggests that *Sutterella* may also influence bone health independent of body composition. This may occur through immune modulation by influencing Th17/Treg balance, which regulate bone resorption and formation,^7^ or microbial metabolite signaling pathways. One potential signaling pathway is the production of indoles and secondary bile acids, which directly act on osteoblast/osteoclast receptors.^45^

Studies suggests that the gut microbiome is interdependent,^44^ therefore may jointly contribute to host health. Therefore, we used Random Forest, a machine learning approach applied previously in microbiome research^31^ to identify combinations of taxa predictive of health outcomes. Our findings indicate that only the gut microbiome at 1 mo was predictive of later bone health outcomes (aBMC), with *Escherichia-Shigella, Haemophilus,* and *Klebsiella* emerging as the top predictive genera at this time point. However, the model explained minimal variance (3.3%), highlighting the complexity of bone health development and suggesting that the gut microbiome plays a limited role in bone health in children.

At 1 wk, 1 yr, 4 yr, and 6 yr of age, the Random Forest models performed poorly, suggesting weak predictive power of available data, likely due to overfitting from data splitting.^30^ We used both linear regression and Random Forest to explore associations between microbial abundance and bone outcomes, allowing us to capture both linear and potential non-linear patterns in the data. Noteworthy, *Escherichia-Shigella* at 1 mo and *Sutterella* at 1 yr consistently ranked among the top predictive genera across both methods, indicating a potential role in bone health that warrants further validation. Larger studies are needed to confirm these findings and refine predictive models.

Our study has notable strengths, including (1) its execution within COPSAC_2010_, a large population-based pregnancy cohort study, (2) assessment of the microbiome at several time points, (3) the bone health outcomes were measured using the gold standard DXA, which ensured reliability of measurements, (4) adjustment for various socioeconomic and lifestyle variables that are likely to influence the association, and (5) the use of advanced statistical methods to assess the microbiome-bone health association.

Our study also has some limitations that warrant discussion. Microbiome analysis was done by 16S rRNA gene amplicon sequencing of the V4 region, which provides detailed characterization of the microbial community but limits detection to the genus level. Therefore, patterns evident at the species or strain taxonomic levels could not be explained. In addition, contrary to shotgun sequencing, the 16S rRNA sequencing is limited to identification and quantification and does not provide information on functional capacity of taxa. Our study focuses on the bacterial aspect of gut microbiome, therefore, any potential role of viruses, fungi, protozoa, etc, on bone health could not be ruled out. The site of fecal sample collection (home vs clinic) was not recorded, which may have introduced variability in microbial composition. However, detailed collection instructions were provided to minimize contamination, and samples were transported to the laboratory with minimal delay. Lastly, microbiome composition has been shown to vary by race;^46^ however, majority of participants were Caucasians, which limits generalizability to other populations.

## Conclusion

Here we explored the longitudinal relationship between the gut microbiome in early life and bone health later in childhood. Although evidence from animal and adult studies suggests that the gut microbiome may play a role in bone homeostasis, we found no consistent relation between the early gut microbiome and bone health outcomes later in childhood. We did observe that *Escherichia-Shigella* at 1 mo and *Sutterella* at 1 yr were associated with bone health at 6 yr, though the small effect sizes and lack of consistency across time points suggest a modest contribution of microbiome composition.

The longitudinal design and careful temporal sequencing in our study reduce the likelihood of confounding or reverse causality, strengthening confidence in our findings. While the early-life microbiome does not appear to play a dominant role in childhood bone health, replication in larger cohorts is required to confirm our findings.

## Supplementary Material

Supplementary_material_v1_3_zjaf108

## Data Availability

The data underlying this article were provided by COPSAC_2010_ under license / by permission. Data will be shared on request to the corresponding author with permission of COPSAC_2010_.
