# Supplementary material for: Gut microbiome in early life and bone health outcomes at age 6: a Danish mother-child cohort study
Source: J Bone Miner Res. 2025 Aug 12;40(11):1278–89. doi: 10.1093/jbmr/zjaf108 (PMC12578300; doi:10.1093/jbmr/zjaf108)
Supplement: Supplementary_material_v1_3_zjaf108 [file supplementary_material_v1_3_zjaf108.docx]

## **Supplementary material**

### **Tables**

**Supplementary Table 1: Summary Study Characteristics**

| **Participant Characteristic** | | | **Number of participants,**  **N=549** | **value** | **Missing,**  **total, %** |
| --- | --- | --- | --- | --- | --- |
| **Mother’s characteristics** | | |  |  |  |
| Age at birth (years) | |  | 549 | 32.4 ± 4.3 |  |
| Education level*, % | | Low | 28 | 5.1 % |  |
|  | | Medium | 361 | 65.8 % |  |
|  | | High | 160 | 29.1 % |  |
| **Child’s characteristics** | |  |  |  |  |
| Sex, % | | boy | 270 | 49.2 % |  |
|  | | girl | 279 | 50.8 % |  |
| Race, % | | Caucasian | 521 | 94.9 % |  |
|  | | non-Caucasian | 28 | 5.1 % |  |
| Gestational age (days) | |  | 549 | 39.9 ± 1.7 |  |
| Duration of exclusive breastfeeding (months) | |  | 549 | 3.5 ± 1.9 |  |
| Mode of delivery, % | | vaginal | 435 | 79.2 % |  |
|  | | planned section | 49 | 8.9 % |  |
|  | | emergency section | 65 | 11.8 % |  |
|  | |  |  |  |  |
| Birth weight (kg) | |  | 549 | 3.5 ± 0.6 |  |
|  |  |  |  |  |  |
| Biannual household income (DKK)**, % | | below 400.000 DKK | 43 | 7.9 % | 8, 1.5% |
|  | | 400.000 - 600.000 DKK | 100 | 18.5 % |  |
|  | | 600.000 - 800.000 DKK | 193 | 35.7 % |  |
|  | | 800.000 - 1.000.000 DKK | 110 | 20.3 % |  |
|  | | above 1.000.000 DKK | 95 | 17.6 % |  |
| **Exposure variables** | |  |  |  |  |
| Microbial reads at: | |  |  |  |  |
| 1 week sampling | |  | 445 | 59382 ± 43199 | 104, 18.9% |
| 1 month sampling | |  | 492 | 55263 ± 41658 | 57, 10.4% |
| 1 year sampling | |  | 508 | 48701 ± 29024 | 41, 7.5% |
| 4 years sampling | |  | 350 | 66292 ± 39628 | 199, 36.2% |
| 6 years sampling | |  | 327 | 40279 ± 13932 | 222, 40.4% |
| **Variables at age 6***** | |  |  |  |  |
| Age at 6-year DXA (years) | |  | 549 | 6.3 ± 0.4 |  |
| Height at DXA (cm) | |  | 549 | 120.9 ± 5.7 |  |
| Bone-free mass (g) | |  | 549 | 13987.5 ± 2090 |  |
| Bone mineral density (mg/cm^2^) | |  | 549 | 0.6 ± 0 |  |
| Bone mineral content (g) | |  | 549 | 544.3 ± 94.4 |  |
| Bone area (cm^2^) | |  | 549 | 963.2 ± 101.3 |  |
| area adjusted bone mineral content (g) | |  | 549 | - 1. ± 36.6 |  |

Values are mean ± standard deviations for continuous variables and percentage and (numbers) for categorical variables. * Level of education: Low = Elementary or College, Medium = Tradesman; High = University; **Biannual income: 1 DDK (Danish krone) ~ 0.14 USD; ***: All DXA scan derived outcomes are presented for Total Body Less Head. Abbreviations: SD (standard deviation); DXA = Dual-Energy X-ray Absorptiometry.

**Supplementary Table 2: Alpha diversity vs bone health outcomes, exploratory model including bone free mass (Observed richness added for sensitivity to compare with PD)**

| **Diversity index** | | **Measure of bone health at age 6** | **1 week**  *n = 445* | **1 month**  *n = 492* | **1 year**  *n = 508* | **4 years**  *n = 350* | **6 years**  *n = 327* |
| --- | --- | --- | --- | --- | --- | --- | --- |
| **MULTIPLE LINEAR REGRESSION; β, (95% CI) p-value, partial R^2^** | | | | | | | |
| Alpha diversity | Faith’s Phylogenetic Diversity (PD) | BMD | -9.517x10^-4^,  (-2.717x10^-3^, 8.132x10^-4^)  0.29, 0.3% | 6.106x10^-4^,  (-1.337x10^-3^, 2.558x10^-3^)  0.54, 0.1% | -1.750x10^-3^,  (-3.394x10^-3^, -1.055x10^-4^)  **0.037*,** 0.8% | -1.370x10^-3^,  (-2.664x10^-3^, -7.700x10^-5^)  **0.038***, 1.3% | -1.046x10^-3^,  (-2.658x10^-3^, 5.665x10^-4^)  0.20, 0.1% |
|  |  | aBMC | -0.96, (-2.83, 0.91)  0.32, 0.03% | 0.51, (-1.56, 2.58)  0.63, 0.1% | -1.30, (-3.05, 0.44)  0.14, 0.4% | -1.82, (-3.21, -0.44)  **0.010***, 1.9% | -1.00, (-2.71, 0.71)  0.25, 0.4% |
|  | Observed Richness | BMD | -3.864x10^-5^,  (-1.879x10^-3^, 1.107x10^-4^)  0.61, 0.05% | 5.324x10^-5^,  (-1.674x10^-4^, 2.739x10^-4^)  0.64, 0.05% | -8.958x10^-5^,  (-2.131x10^-4^, 3.394x10^-5^)  0.16, 0.41% | -1.208x10^-4^,  (-2.073x10-4, -3.428x10^-5^)  **0.006****, 2.2% | -3.667x10^-5^,  (-1.376x10^-4^, 6.423x10^-5^)  0.48, 0.16% |
|  |  | aBMC | -0.04, (-0.20, 0.12)  0.62, 0.06% | 0.05, (-0.18, 0.29)  0.67, 0.04% | -0.07, (-0.20, 0.07)  0.33, 0.19% | -0.15, (-0.25, -0.06)  **0.001****, 3.10% | -0.03, (-0.14, 0.08)  0.58, 0.10% |
| **PERMANOVA (for all participants); F-score, partial R^2^, p-value** | | | | | | | |
| Beta diversity | Weighted UniFrac | BMD | 1.74, 0.3%, 0.10 | 0.88, 0.2%, 0.51 | 0.89, 0.2%, 0.52 | 1.63, 0.5%, 0.09 | 1.92, 0.6%, **0.026*** |
|  |  | aBMC | 1.11, 0.3%, 0.30 | 0.63, 0.1%, 0.74 | 1.01, 0.2%, 0.41 | 0.89, 0.3%, 0.50 | 1.22, 0.4%, 0.21 |
|  | Unweighted UniFrac | BMD | 1.28, 0.3%, 0.15 | 1.20, 0.2%, 0.21 | 0.960, 0.2%, 0.51 | 1.33, 0.4%, 0.07 | 1.25, 0.4%, 0.10 |
|  |  | aBMC | 0.95, 0.2%, 0.49 | 0.73, 0.1%, 0.86 | 0.97, 0.2%, 0.48 | 1.02, 0.3%, 0.39 | 1.07, 0.3%, 0.31 |

Abbreviations: **BMD**, bone mineral density; **aBMC**, area-adjusted bone mineral content. Associations between alpha diversity and bone health outcomes were assessed using linear regression, while associations for beta diversity were evaluated using permutational analysis of variance (PERMANOVA). Models were adjusted for child’s sex, race, socio-economic status of the family, and age, height, and bone-free mass at the time of the DXA scan, and log (library size). Partial R² values indicate the proportion of variance in the bone health outcome uniquely explained by the diversity measure after accounting for other covariates in the model. Summary statistics are presented for each time point, with nominally significant results denoted by ***** for p < 0.05 and ****** for p < 0.01.

**Supplementary Table 3: Summary results for LIMMA (bacterial taxa vs categorical bone health outcomes)**

|  | **Bone measure – tertiles *^c^*** | | | |
| --- | --- | --- | --- | --- |
| **Bacterial taxa** | **BMD (LogFC, p-value)** | **Adjusted p-value (BH)** *^a^* | **aBMC (LogFC, p-value)** | **Adjusted p-value (BH)** *^a^* |
| **1 week (*n=17*)** *^b^* | **1/17 *with p<0.05*** | **0/17 *with p.adj<0.05*** | **1/17 *with p<0.05*** | **0/17 *with p.adj<0.05*** |
| *Parabacteroides* | 1.806, **0.046*** | 0.461 | -0.043, 0.924 | 0.924 |
| *Klebsiella* | 0.760, 0.400 | 0.808 | 0.997, **0.028*** | 0.337 |
| **1 month (n=21)** *^b^* | **1/21 *with p<0.05*** | **1/21 *with p.adj<0.05*** | **1/21 *with p.adj<0.05*** | **0/21 *with p.adj<0.05*** |
| *Escherichia-Shigella* | -1.651, **0.002**** | **0.038*** | -0.921, **0.021*** | 0.416 |
| **1 year (*n=52*)** *^b^* | **0/52 *with p<0.05*** | **1/52 *with p<0.05*** | **3/52 *with p<0.05*** | **1/52 *with p<0.05*** |
| *Parasutterella* | 0.923, 0.064 | 0.983 | 0.847, **0.024*** | 0.488 |
| *[Eubacterium] hallii group* | 0.604, 0.150 | 0.983 | 0.796, **0.011*** | 0.575 |
| *Sutterella* | -0.447, 0.384 | 0.983 | -0.766, **0.046*** | 0.762 |
| **4 years (*n=67*)** *^b^* | **0/67 *with p<0.05*** | **0/67 *with p<0.05*** | **8/67 *with p<0.05*** | **0/67 *with p<0.05*** |
| *Parabacteroides* | -0.428, 0.433 | 0.997 | -0.806, **0.048*** | 0.311 |
| *Oscillibacter* | -0.503, 0.260 | 0.997 | -0.808, **0.015*** | 0.311 |
| *Lachnobacterium* | -0.834, 0.114 | 0.997 | -1.038, **0.008*** | 0.311 |
| *Family XIII AD3011 group* | -0.949, 0.064 | 0.997 | -0.901, **0.019*** | 0.311 |
| *Christensenellaceae R-7 group* | -0.451, 0.423 | 0.997 | -0.969, **0.020*** | 0.311 |
| *Odoribacter* | -0.825, 0.084 | 0.997 | -0.799, **0.025*** | 0.311 |
| *Pseudoflavonifractor* | -0.791, 0.107 | 0.997 | -0.800, **0.029*** | 0.311 |
| *Colidextribacter* | -0.476, 0.313 | 0.997 | -0.748, **0.033*** | 0.311 |
| *Sporobacter ^d^* | *NA* | *NA* | -1.400, **0.0004***** | **0.024*** |
| *Christensenellaceae R-7 group ^d^* | *NA* | *NA* | -1.333, **0.001**** | **0.028*** |
| *Oscillibacter ^d^* | *NA* | *NA* | -1.036, **0.001**** | **0.028*** |
| **6 years (*n=72*)** *^b^* | **0/72 *with p.adj<0.05*** | **0/72 *with p.adj<0.05*** | **0/72 *with p.adj<0.05*** | **0/72 *with p.adj<0.05*** |
| *NA* | *NA* | *NA* | *NA* | *NA* |

Only taxa with at least one p-value < 0.05 for either bone health outcomes are reported. *^a^*Benjamini-Hochberg: method used to adjust for multiple testing. *^b^*Taxa of interest were obtained based on most prevalent and abundant taxa (Prevalence = 25%*(1e-4/Mean Relative Abundance)^0.5). *^c^*High tertiles were compared to low tertiles. *^d^*Genera with differential abundance between low and medium aBMC tertiles. *****statistically significant at p<0.05, ******statistically significant at p<0.01, *******statistically significant at p<0.001. LogFC: Log-fold change is the log-ratio of the association of the relative abundance of the microbe per SD increase in the bone outcome

#### **Supplementary Table 4: Summary results for linear regression analysis (genera vs continuous bone health outcomes), model including bone free mass**

| **Bacteria genera with low p-values (n=230)** | **BMD / gcm^-2^** | | **aBMC / g** | |
| --- | --- | --- | --- | --- |
|  | **β [95% CI], p-value, partial R^2^** | **Adjusted p-value** | **β [95% CI], p-value, partial R^2^** | **Adjusted p-value** |
| **1 week (*n=17*)** | **0/17 *with p<0.05*** | **0/17 *with p.adj<0.05*** | **0/17 *with p<0.05*** | **0/17 *with p.adj<0.05*** |
| *NA* | *NA* | *NA* | *NA* | *NA* |
| **1 month (*n=21*)** | **2/21 *with p<0.05*** | **0/21 *with p.adj<0.05*** | **1/21 *with p<0.05*** | **0/21 *with p.adj<0.05*** |
| *Escherichia-Shigella* | -1.455x10^-3^ [-2.409x10^-3^, -4.996x10^-4^], **0.003****, 1.8% | 0.061 | -1.40 [-2.42, -0.38], **0.007****, 1.5% | 0.147 |
| *Klebsiella* | 9.254x10^-4^ [8.353x10^-5^, 1.767x10^-3^], **0.031***, 1.0% | 0.328 | 0.80 [-0.09, 1.70], 0.079, 0.6% | 0.554 |
| **1 year (*n=52*)** | **5/52 *with p<0.05*** | **0/52 *with p.adj<0.05*** | **3/52 *with p<0.05*** | **1/52 *with p.adj<0.05*** |
| *Sutterella* | -1.893x10^-3^ [-3.035x10^-3^, -7.506x10^-4^], **0.001****, 2.1% | 0.063 | -2.16 [-3.37, -0.95], **0.0005*****, 2.4% | **0.025*** |
| *[Eubacterium] hallii group* | 1.727x10^-3^ [5.198x10^-4^, 2.945x10^-3^], **0.006****, 1.5% | 0.143 | 1.69 [0.40, 2.99], **0.010***, 1.3% | 0.267 |
| *Monoglobus* | 1.794x10^-3^ [2.401x10^-4^, 3.347x10^-3^], **0.024***, 1.0% | 0.209 | 1.66 [0.02, 3.31], **0.048***, 0.8% | 0.692 |
| *Bifidobacterium* | 1.690x10^-3^ [2.220x10^-4^, 3.157x10^-3^], **0.024***, 1.0% | 0.209 | 1.39 [-0.17, 2.95], 0.081, 0.6% | 0.692 |
| *Parasutterella* | 1.213x10^-3^ [5.889x10^-5^, 2.368x10^-3^], **0.039***, 0.8% | 0.256 | 1.12 [-0.10, 2.35], 0.072, 0.6% | 0.692 |
| *Oscillibacter* | -1.703x10^-3^ [-3.177x10^-3^, -2.294x10^-4^], **0.024***, 1.0% | 0.209 | -1.05 [-2.62, 0.52], 0.189, 0.3% | 0.782 |
| *Flavonifractor* | -1.780x10^-3^ [-3.464x10^-3^, -9.531x10^-5^], **0.038***, 0.8% | 0.256 | -1.17 [-2.96, 0.62], 0.200, 0.3% | 0.782 |
| **4 years (*n=67*)** | **6/67 *with p<0.05*** | **0/67 *with p.adj<0.05*** | **8/67 *with p<0.05*** | **0/67 *with p.adj<0.05*** |
| *Oscillibacter* | -2.518x10^-3^ [-4.547x10^-3^, -4.882x10^-4^], **0.015***, 1.7% | 0.298 | -3.0 [-5.18, -0.84], **0.007****, 2.1% | 0.146 |
| *Family XIII AD3011 group* | -2.164x10^-3^ [-3.948x10^-3^, -3.809x10^-4^], **0.018***, 1.6% | 0.298 | -2.58 [-4.49, -0.67], **0.008****, 2.0% | 0.146 |
| *Colidextribacter* | -2.111x10^-3^ [-4.015x10^-3^, -2.062x10^-4^], **0.030***, 1.4% | 0.334 | -2.53 [-4.57, -0.50], **0.015***, 1.7% | 0.201 |
| *Clostridia g.* | -1.978x10^-3^ [-4.245x10^-3^, 2.892x10^-4^], 0.087, 0.9% | 0.648 | -2.54 [-4.97, -0.12], **0.040***, 1.2% | 0.334 |
| *Odoribacter* | -2.549x10^-3^ [-4.219x10^-3^, -8.787 x10^-4^], **0.003****, 2.6% | 0.193 | -2.43 [-4.22, -0.63], **0.008****, 2.0% | 0.146 |
| *Lachnobacterium* | -1.961x10^-3^ [-3.589x10^-3^, -3.328x10^-4^], **0.018***, 1.6% | 0.298 | -2.34 [-4.081, -0.59], **0.008****, 2.0% | 0.146 |
| *Incertae Sedis* | -1.713x10^-3^ [-3.524x10^-3^, 9.721x10^-5^], 0.064, 1.0% | 0.532 | -2.18 [-4.11, -0.24], **0.028***, 1.4% | 0.266 |
| *Pseudoflavonifractor* | -1.943x10^-3^ [-3.606x10^-3^, -2.791x10^-4^], **0.022***, 1.5% | 0.298 | -2.10 [-3.89, -0.32], **0.021***, 1.6% | 0.233 |
| **6 years (*n=72*)** | **3/72 *with p<0.05*** | **0/72 *with p.adj<0.05*** | **1/72 *with p<0.05*** | **0/72 *with p.adj<0.05*** |
| *Terrisporobacter* | 2.132x10^-3^ [1.230x10^-4^, 4.140x10^-3^], **0.038***, 1.3% | 0.704 | 1.34 [-0.80, 3.48], 0.218, 0.5% | 0.844 |
| *[Eubacterium] eligens group* | 1.985x10^-3^ [1.583x10^-4^, 3.812x10^-3^], **0.033***, 1.4% | 0.704 | 1.94 [0.003, 3.88], 0.050, 1.2% | 0.844 |
| *Prevotella* | -1.165x10^-3^ [-2.391x10^-3^, 6.163x10^-5^], 0.063, 1.0% | 0.704 | -1.67 [-2.97, -0.38], **0.011***, 2.0% | 0.817 |
| *Pseudoflavonifractor* | -2.464x10^-3^ [-4.659x10^-3^, -2.694x10^-4^], **0.028***, 1.5% | 0.704 | -1.67 [-4.00, 0.67], 0.161, 0.6% | 0.844 |

Regression estimates show the change in bone outcome per centered-log ratio unit increase in each genus abundance. Only genera with p < 0.05 (without multiple testing adjustment) for either bone outcome measures are shown. For every genus with significant association with either aBMC or BMD, the corresponding results for the other bone outcome is shown. The covariates adjusted for included child’s sex, race, socio-economic status, and age, height, and bone free mass at DXA visit. Nominal significant results are indicated by ***** for p<0.05, ****** for p<0.01, and ******* for p<0.001. *p.adj* shows p-values adjusted for multiple testing by Benjamini-Hochberg (FDR threshold of 0.05)

### **Figures**


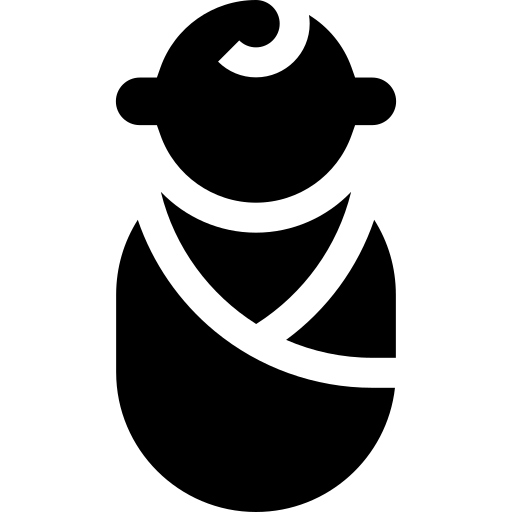

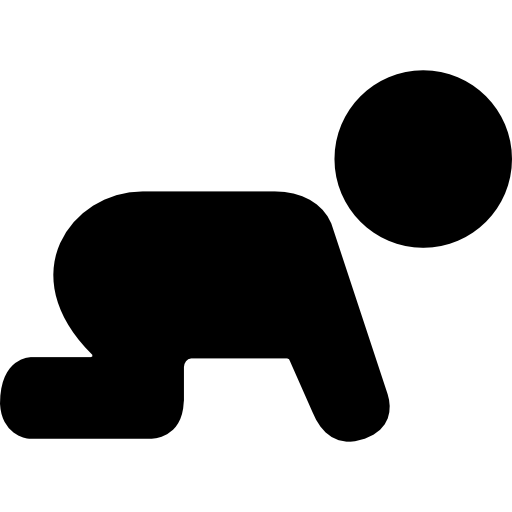

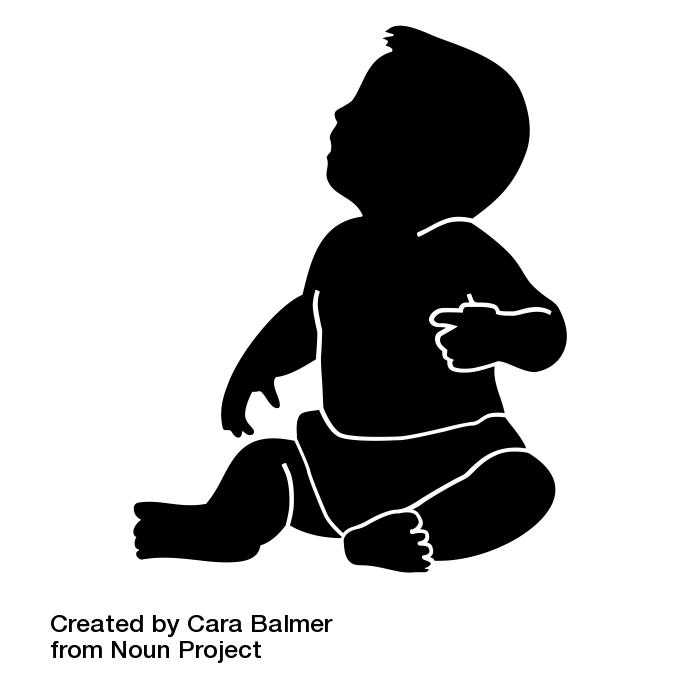


1 week

1 month

1 year

4 years


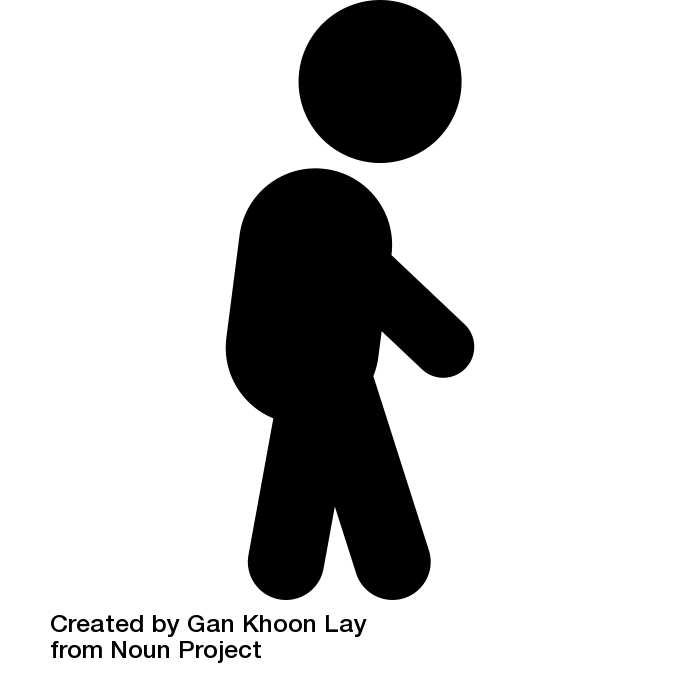

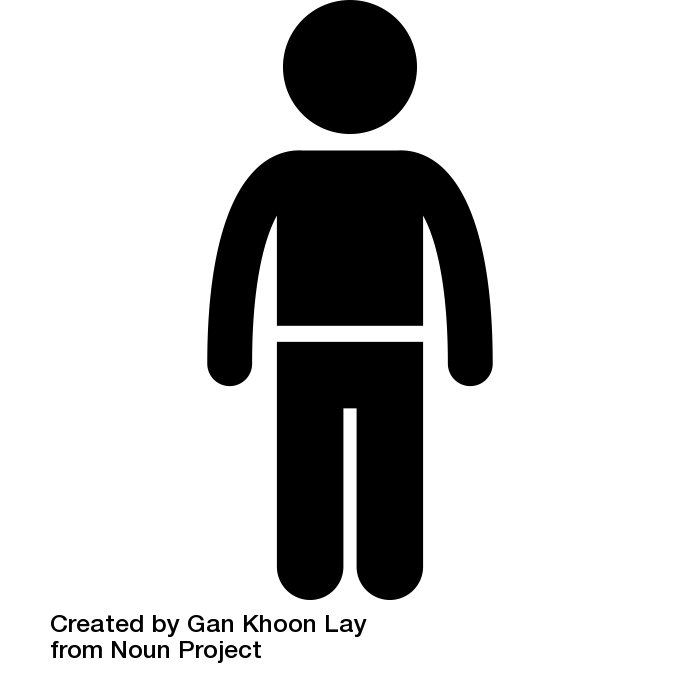


6 years

**Microbiome analysis**

*n=445*

*n=492*

*n=508*

*n=350*

*n=552*

*by 16s rRNA sequencing of V4 region*


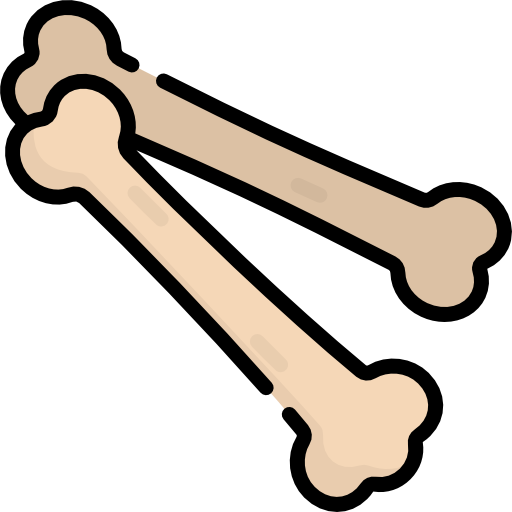


**BMD / area-adjusted (a)BMC**

**EXPOSURE**

**OUTCOME**

*by Dual-energy X-ray Absorptiometry (DXA)*

**α-diversity** *(Observed Richness/PD)*

**β-diversity**

**Abundance/Prevalence**


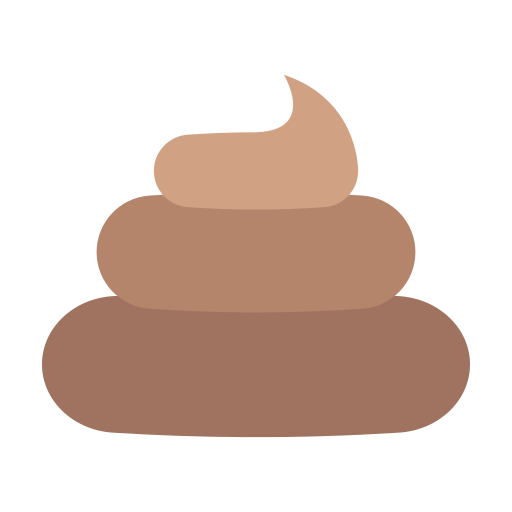


*(UniFrac(w/unweighted))*

PERMANOVA

Prevalence/Abundance plot

Linear Regression

Linear Regression (CLR)

Random Forest (CLR)

Differential abundance (LIMMA-ALR) test

**Supplementary Figure 1: Study overview**. Exposures, assessed by 16s rRNA v4 region sequencing were assessed at all five time points. Bone health outcome was assessed as continuous and categorical variables. The microbiome (characterized by α-diversity, β-diversity, and abundance/prevalence) was associated with bone health outcomes assessed at age 6. Abbreviations: CLR: Centered Log-Ratio transformed abundances used; LIMMA-ALR: Linear Model for Microarray and Omics Data – Additive Log-Ratio; PD: Faith’s Phylogenetic Diversity index


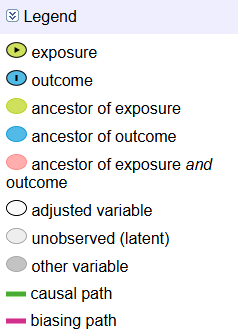


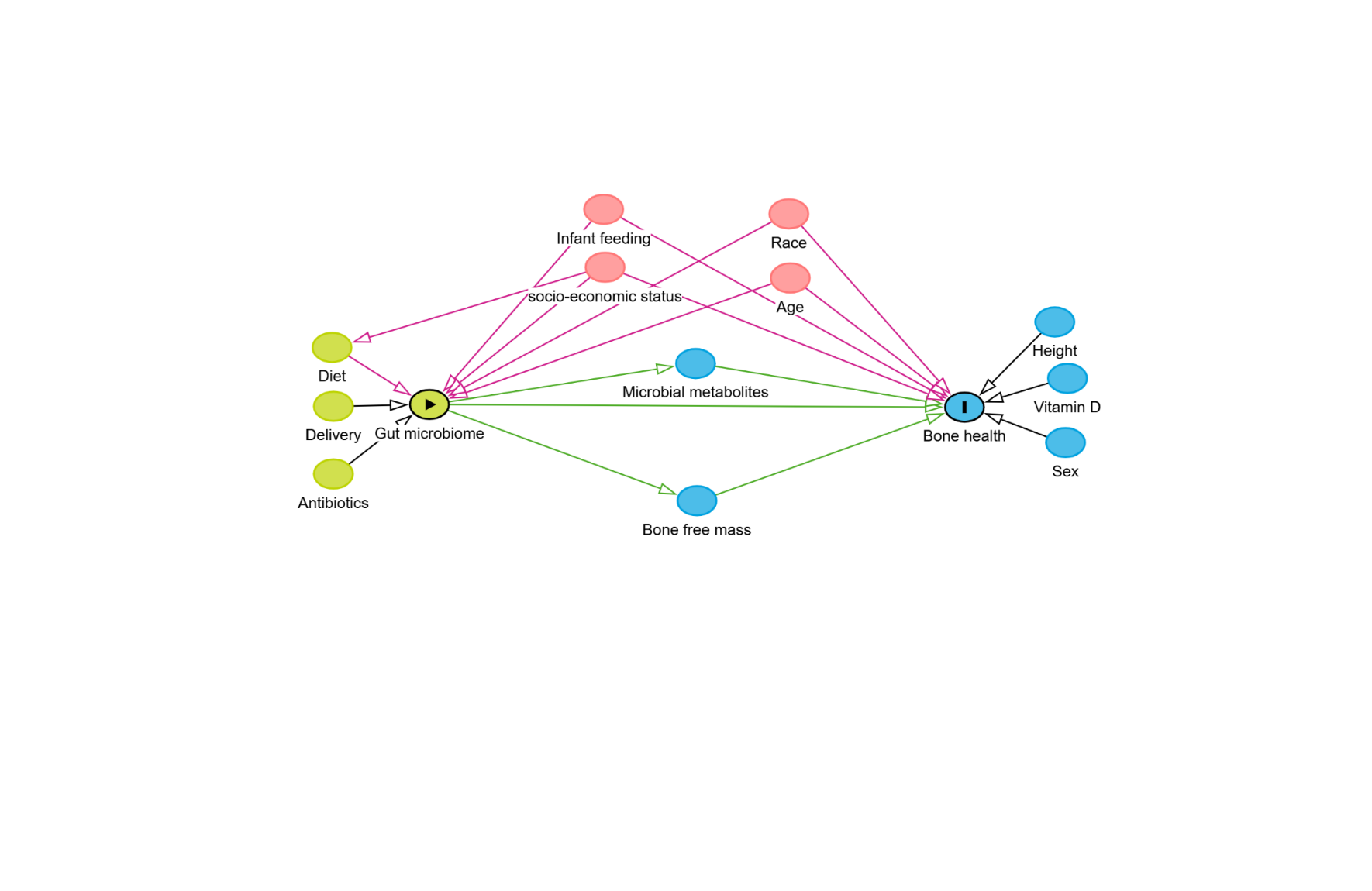


**Supplementary Figure 2: Directed Acyclic Graph. ;** represents **Exposure variable**, **I**; represents outcome variable. Potential confounders indicated by pink circles. Ancestor of exposure in green circles. Ancestor of outcome in blue circles. Green lines indicate causal path.


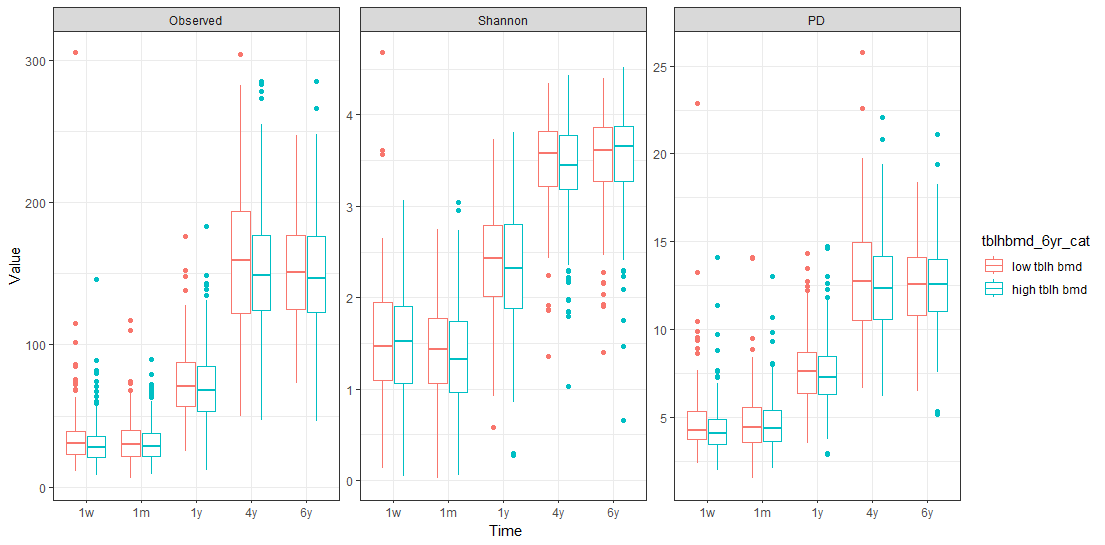

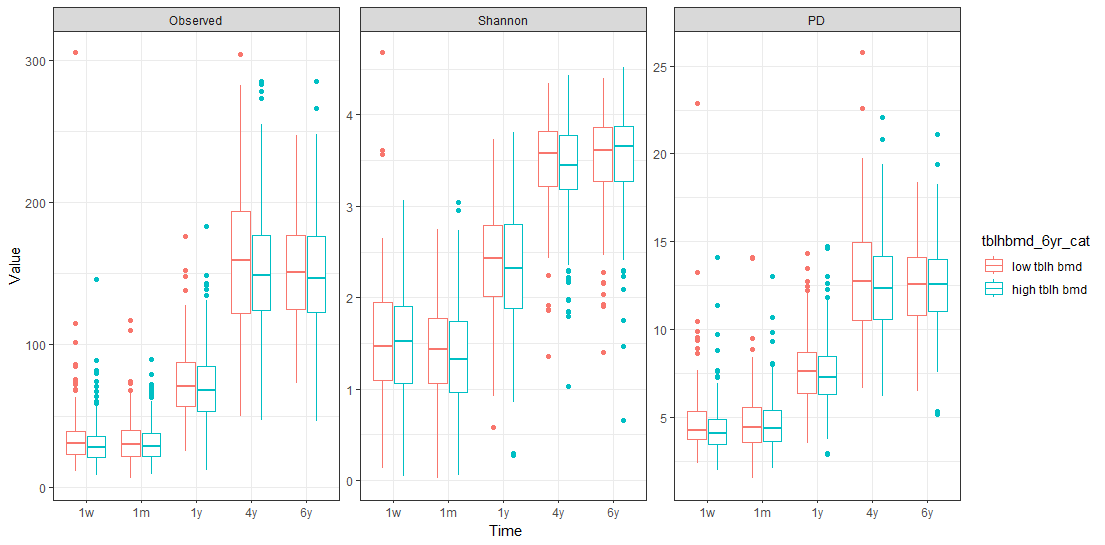


*P=0.072*

*P=0.274*

***P=0.0.043****

*P=0.091*

*P=0.932*

*P=0.085*

*P=0.343*

***P=0.013****

*P=0.434*

*P=0.928*

Time

**Supplementary Figure 3a: Alpha diversity patterns with low/high BMD.** Comparison of alpha diversity between groups of bone health. Bone outcome grouped by median split into low (red) vs high (blue) BMD. Alpha diversity measures; Observed richness and PD are shown for comparison. ANOVA used to test for difference between groups. Abbreviations: PD; Faith’s phylogenetic diversity index, TBLH BMD; Total body less head bone mineral density.


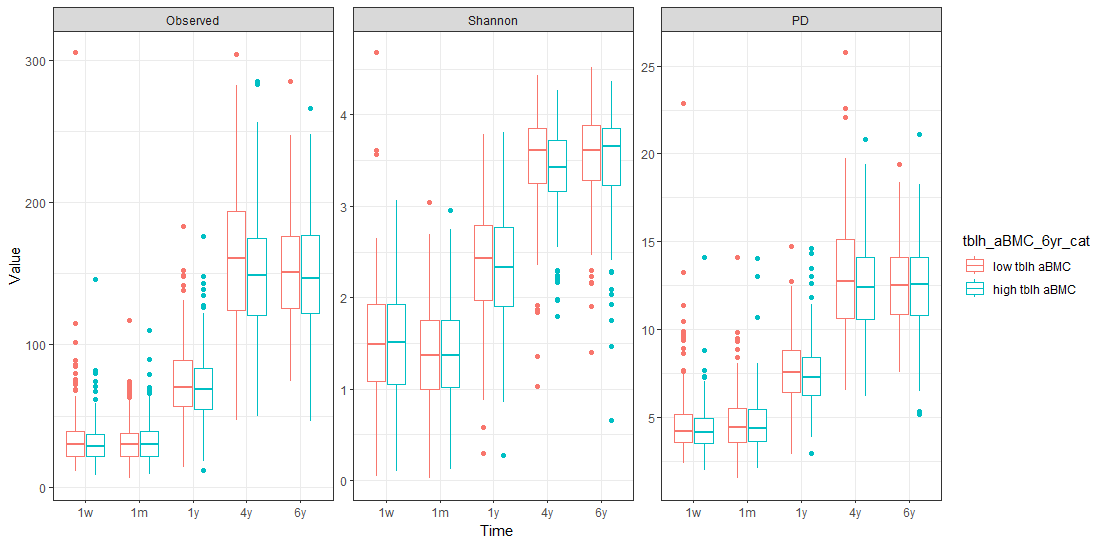

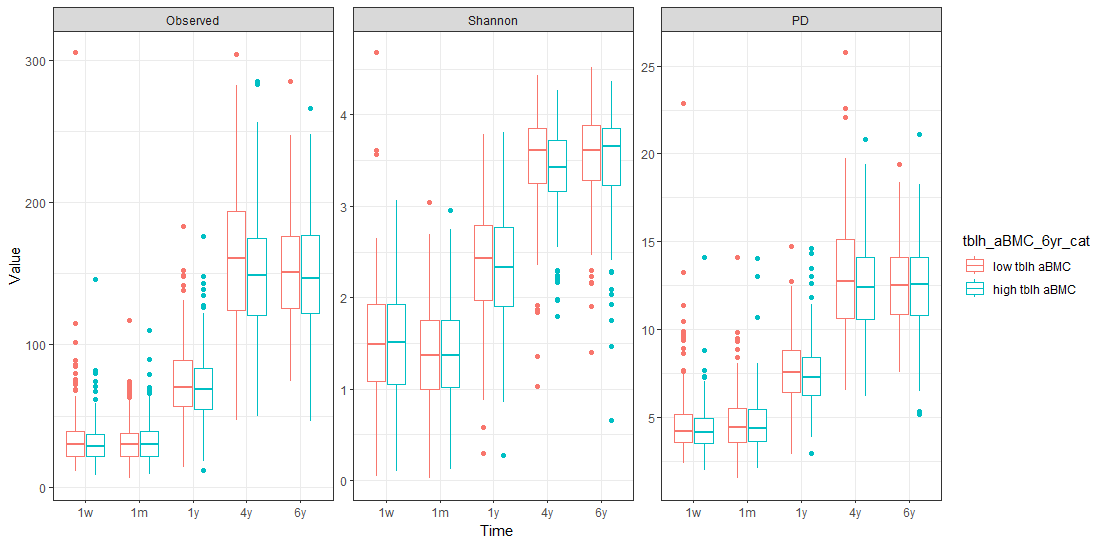


*P=****0.005*****

*P=0.113*

*P=0.780*

*P=0.119*

*P=0.725*

*P=0.063*

*P=0.061*

*P=0.646*

*P=0.069*

*P=0.627*

Time

**Supplementary Figure 3b: Alpha diversity patterns with low/high aBMC.** Comparison of alpha diversity between groups of bone health. Bone outcome grouped by median split into low (red) vs high (blue) aBMC. Alpha diversity measures; Observed richness and PD are shown for comparison. ANOVA used to test for difference between groups. Abbreviations: PD; Faith’s phylogenetic diversity index, TBLH aBMC; Total body less head area-adjusted bone mineral content.


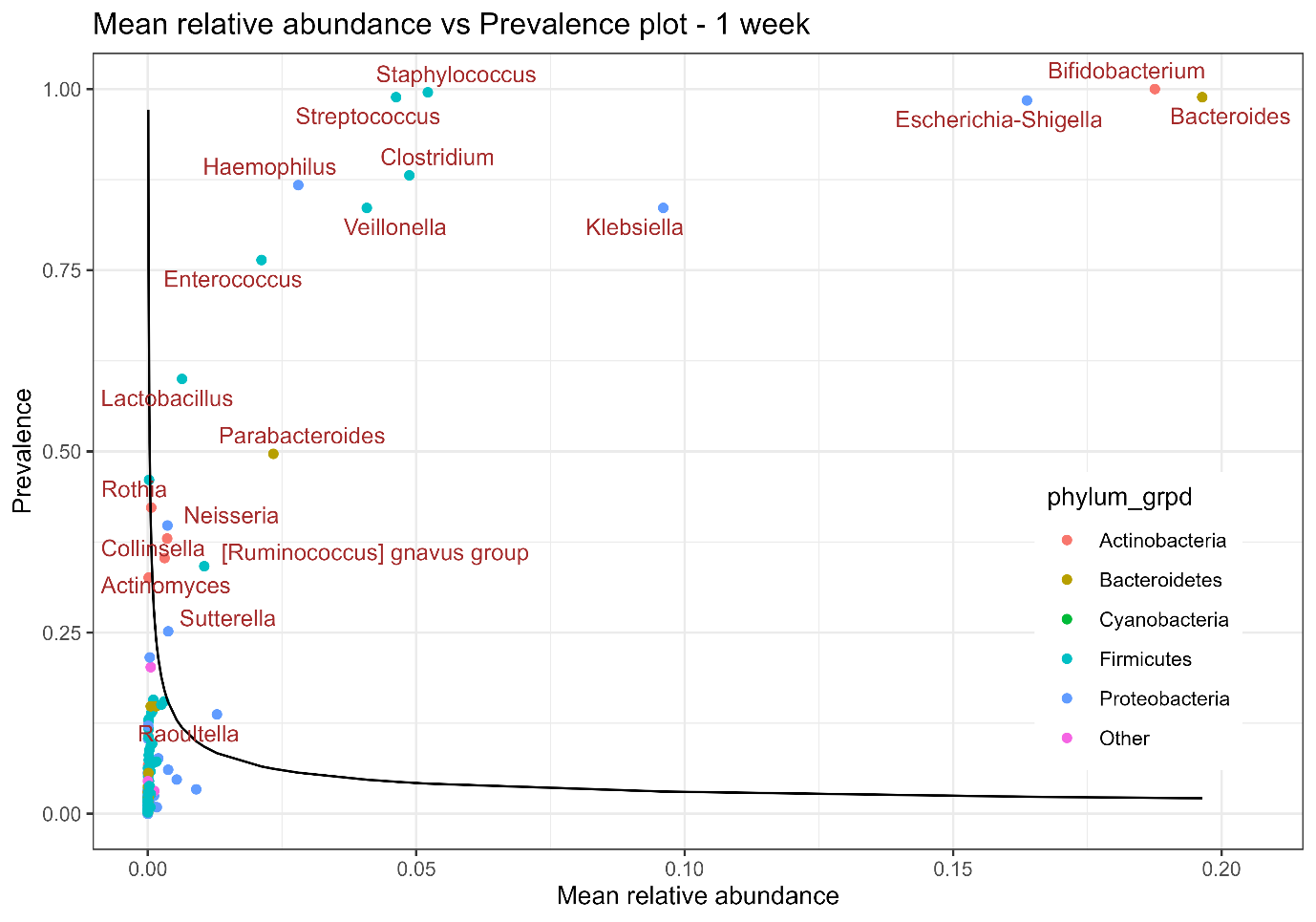


**Supplementary Figure 4a: Bacterial prevalence vs abundance plots at 1 week.** Comparison of mean relative abundance and prevalence for all genera identified in the 16S sequencing data of 1-week-olds (n=445). The most common genera above the curve line (Prevalence = 25%*(1e-4/Mean Relative Abundance)^0.5) are annotated and were selected for downstream analysis.


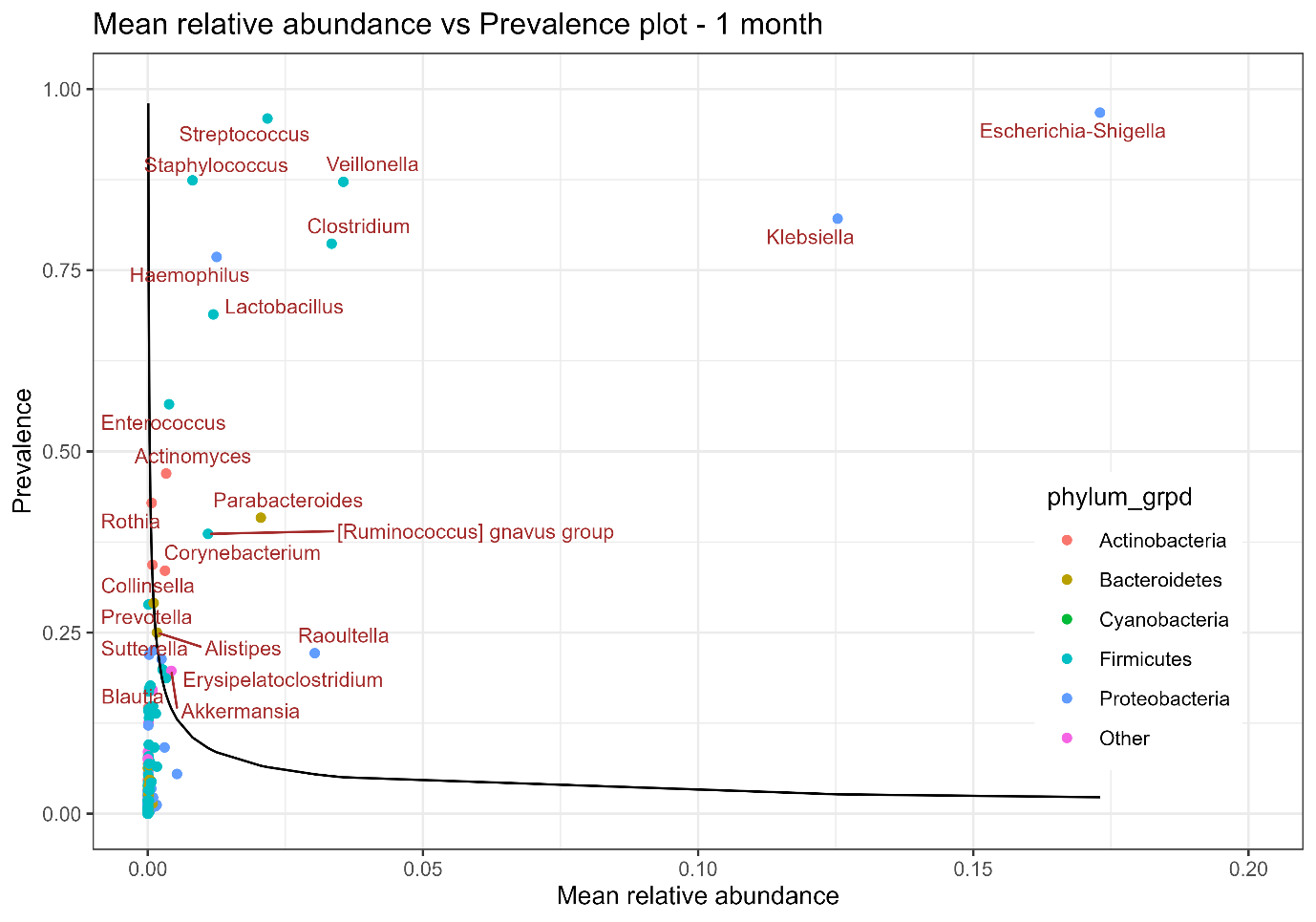


**Supplementary Figure 4b: Bacterial prevalence vs abundance plots at 1 month.** Comparison of mean relative abundance and prevalence for all genera identified in the 16S sequencing data of 1-month-olds (n=492). The most common genera above the curve line (Prevalence = 25%*(1e-4/Mean Relative Abundance)^0.5) are annotated and were selected for downstream analysis.


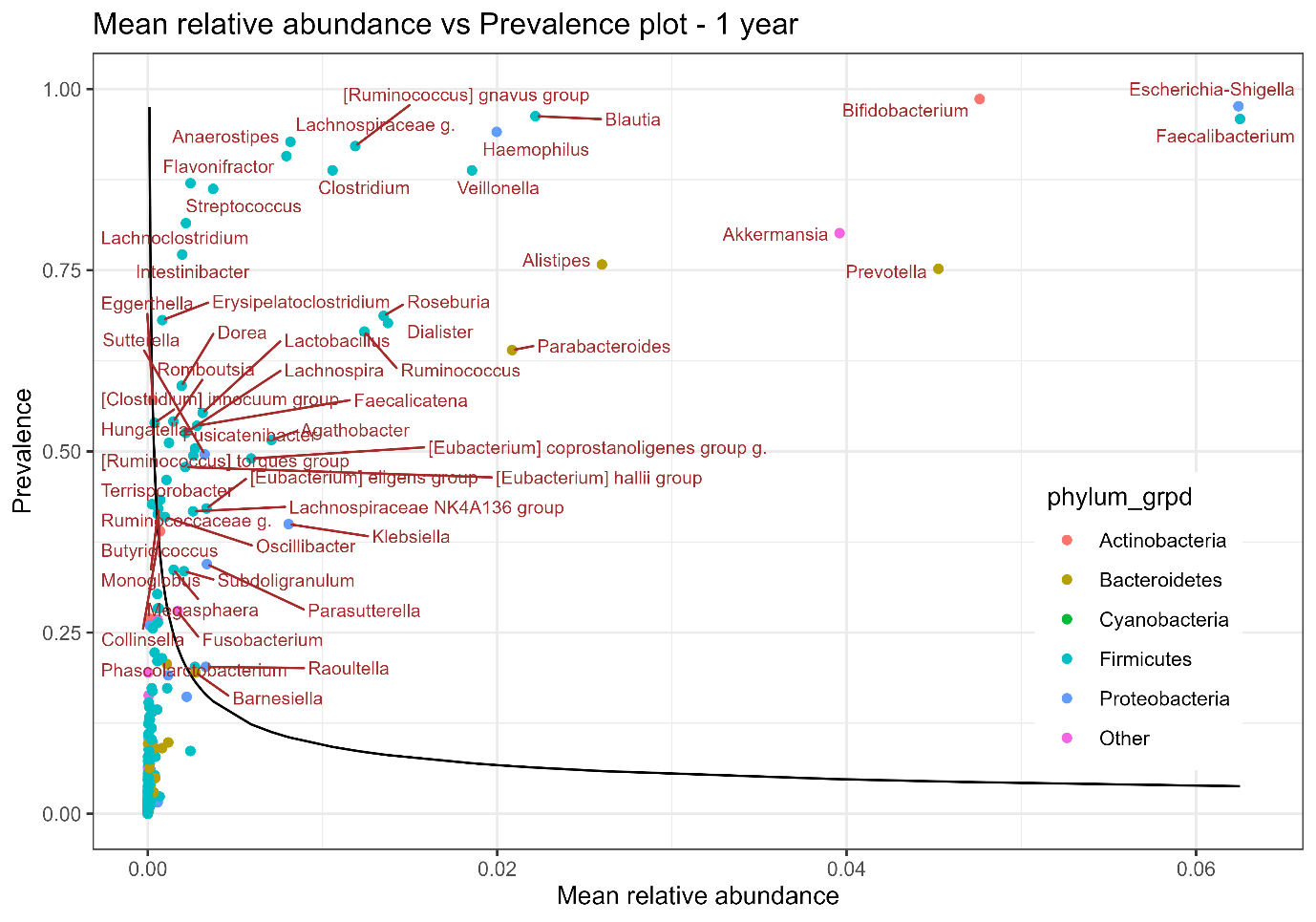


**Supplementary Figure 4c: Bacterial prevalence vs abundance plots at 1 year.** Comparison of mean relative abundance and prevalence for all genera identified in the 16S sequencing data of 1-year-olds (n=508). The most common genera above the curve line (Prevalence = 25%*(1e-4/Mean Relative Abundance)^0.5) are annotated and were selected for downstream analysis.


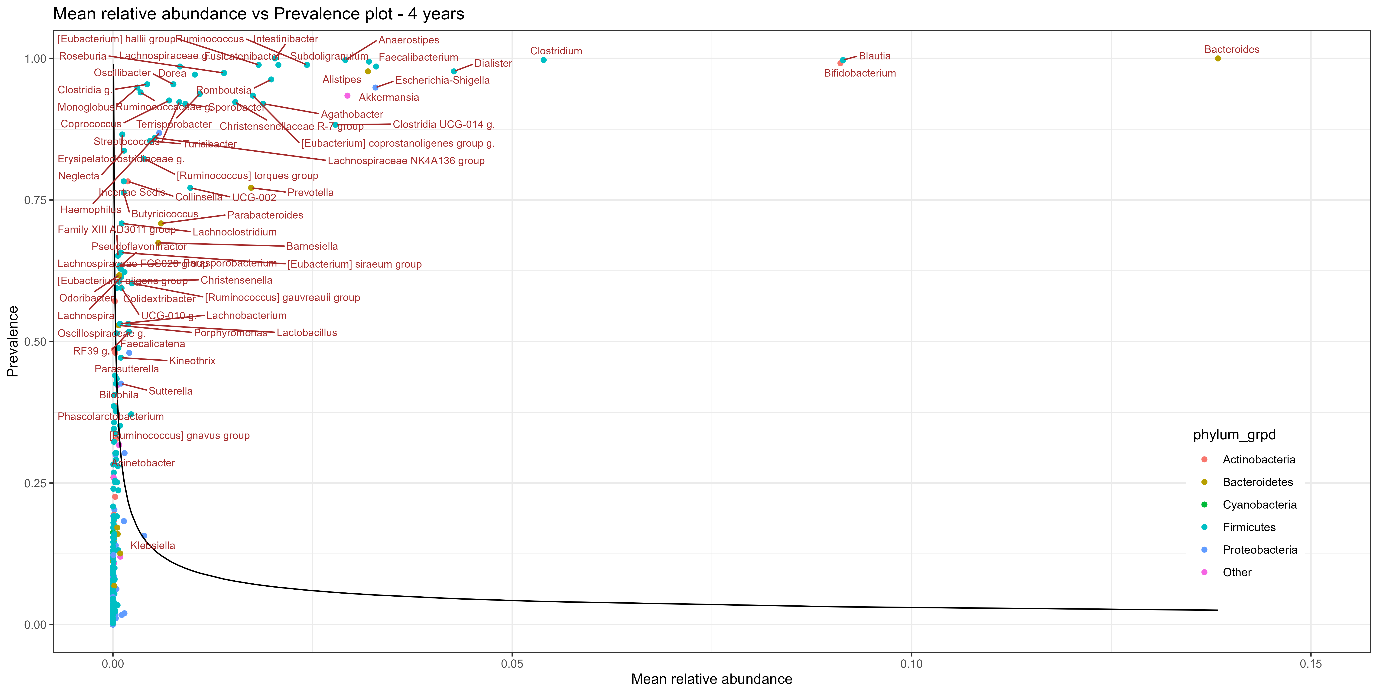


**Supplementary Figure 4d: Bacterial prevalence vs abundance plots at 4 years.** Comparison of mean relative abundance and prevalence for all genera identified in the 16S sequencing data of 4-year-olds (n=350). The most common genera above the curve line (Prevalence = 25%*(1e-4/Mean Relative Abundance)^0.5) are annotated and were selected for downstream analysis.


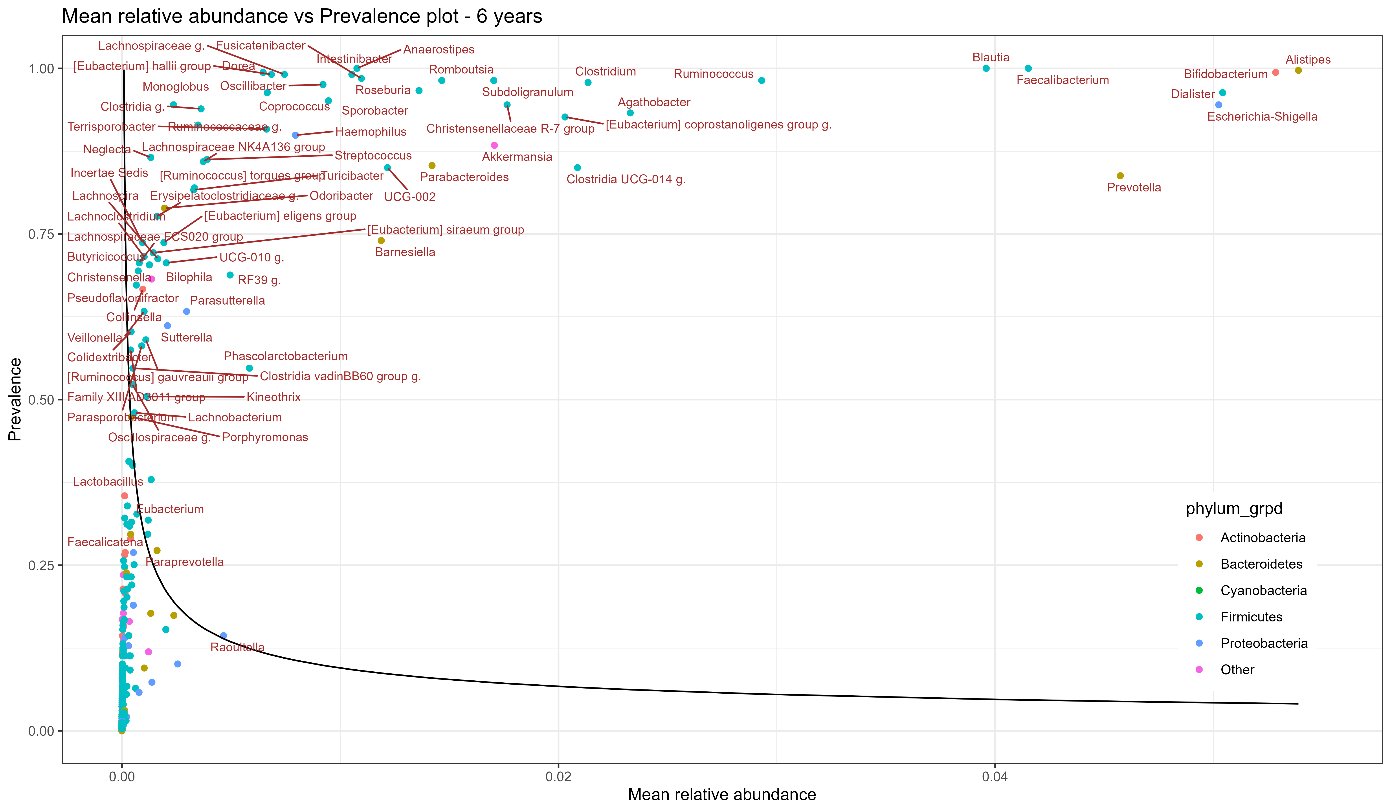


**Supplementary Figure 4e: Bacterial prevalence vs abundance plots at 6 years.** Comparison of mean relative abundance and prevalence for all genera identified in the 16S sequencing data of 6-year-olds (n=327). The most common genera above the curve line (Prevalence = 25%*(1e-4/Mean Relative Abundance)^0.5) are annotated and were selected for downstream analysis.


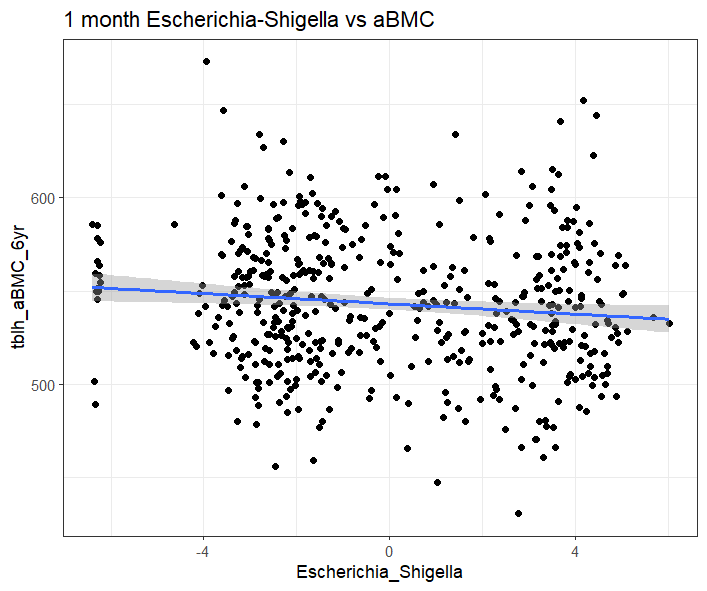


**Supplementary Figure 5a: Linear regression for 1-month taxa vs aBMC at 6 years**. Abundance data for taxa is centered log-ratio transformed. aBMC in grams. TBLH aBMC: Total body less head area-adjusted bone mineral content.


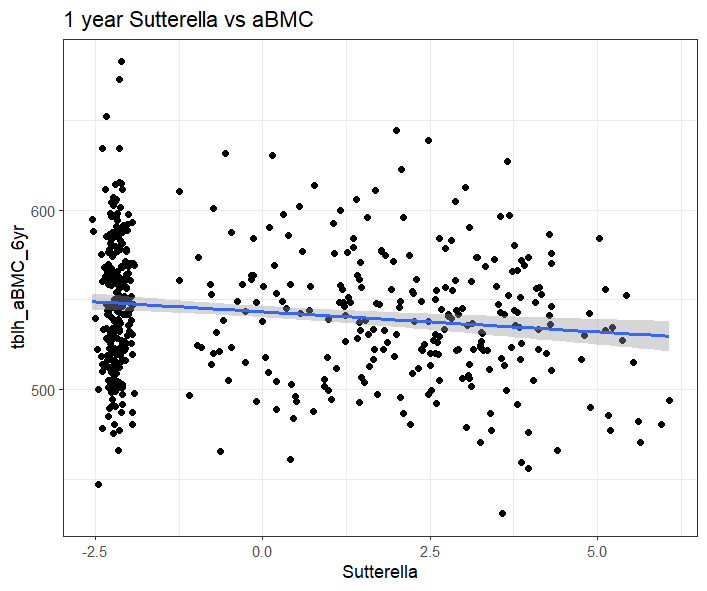

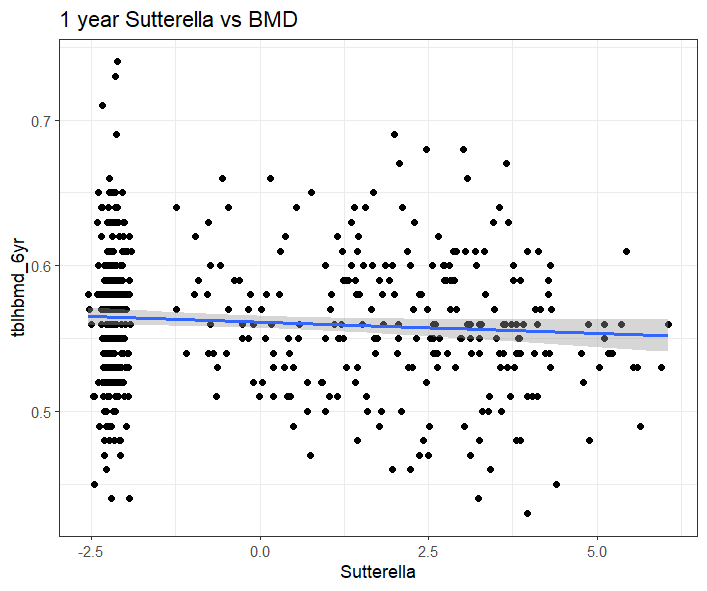


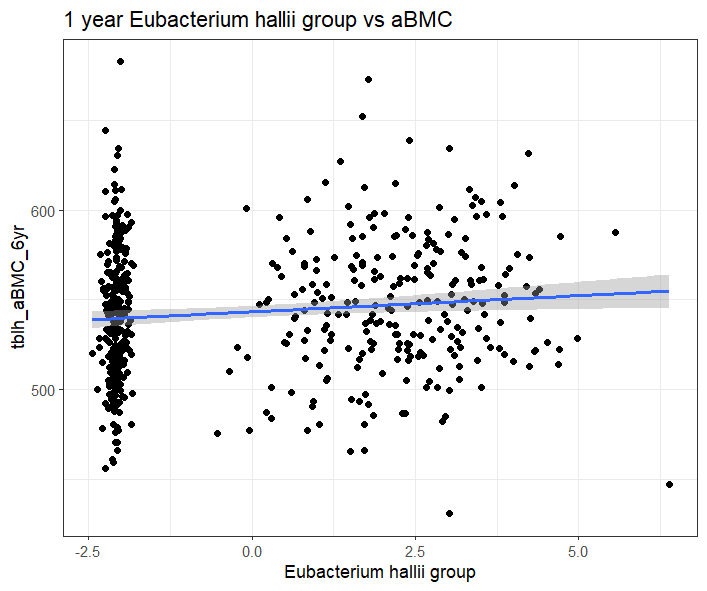

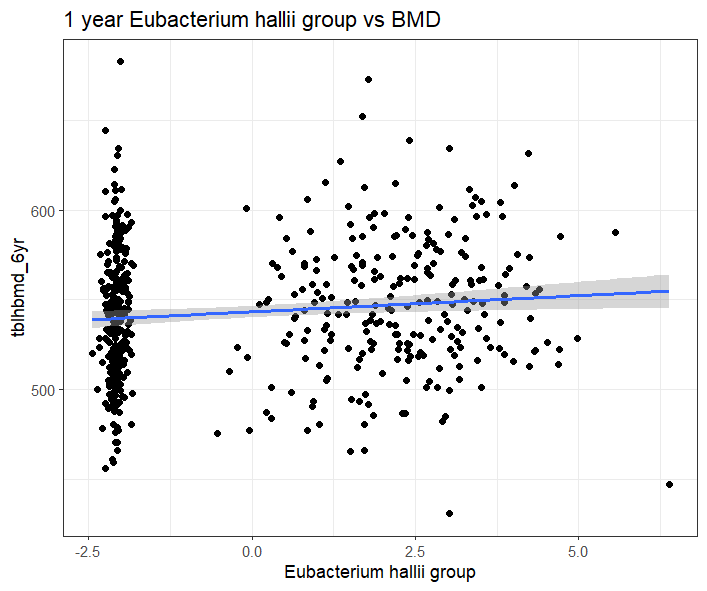


**Supplementary Figure 5b: Linear regression for 1-year taxa vs aBMC at 6 years**. Abundance data for taxa is centered log-ratio transformed. aBMC in grams. TBLH aBMC: Total body less head area-adjusted bone mineral content.

**
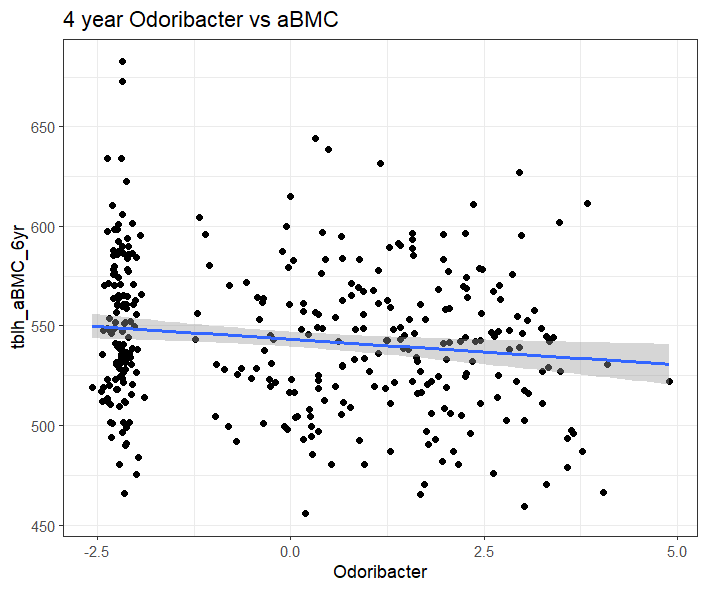

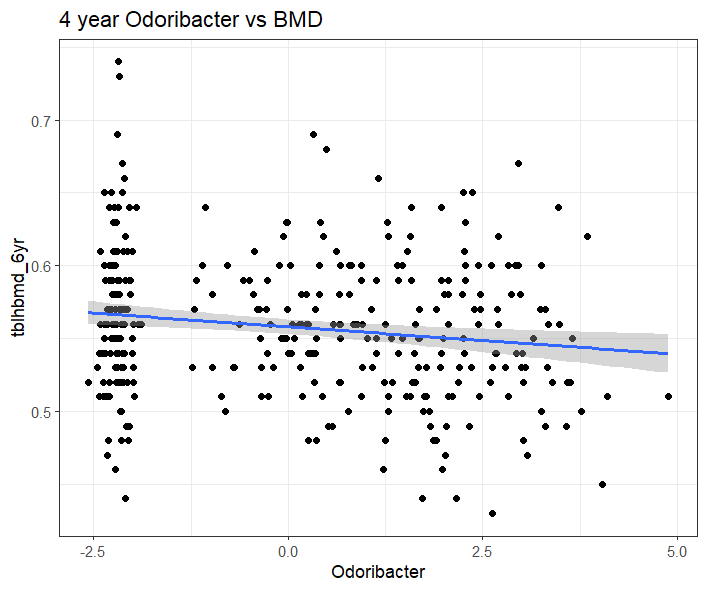
**

**
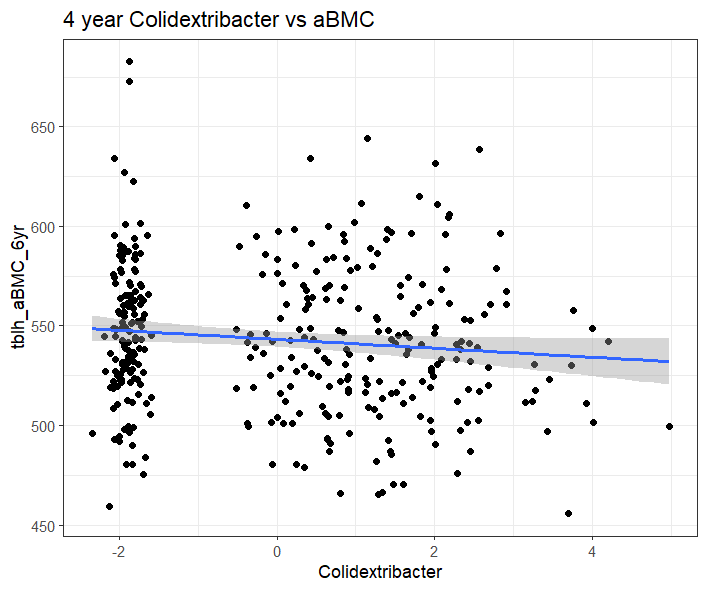

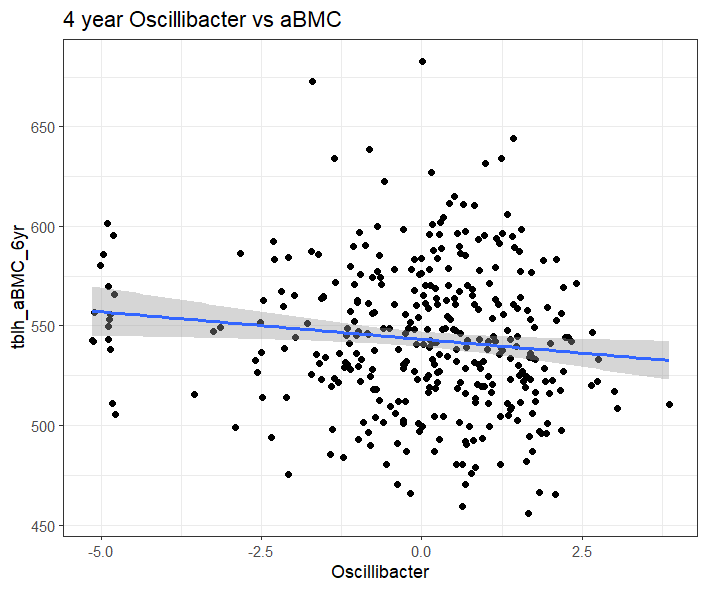
**

**
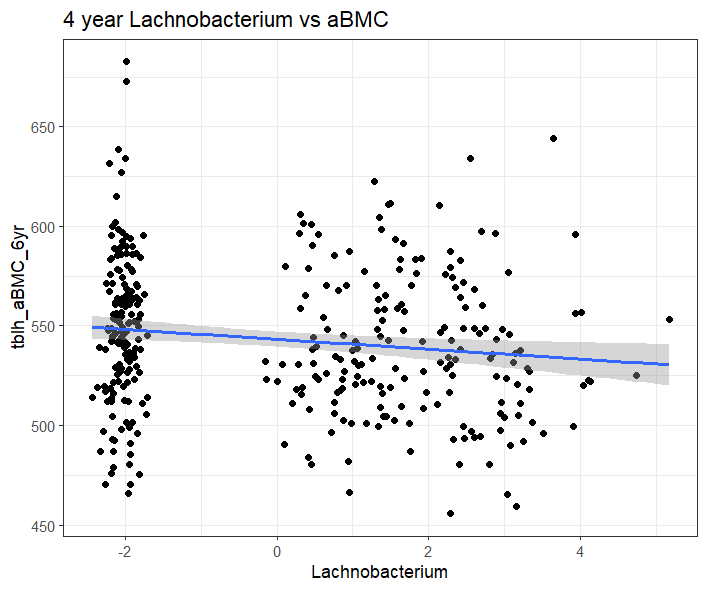

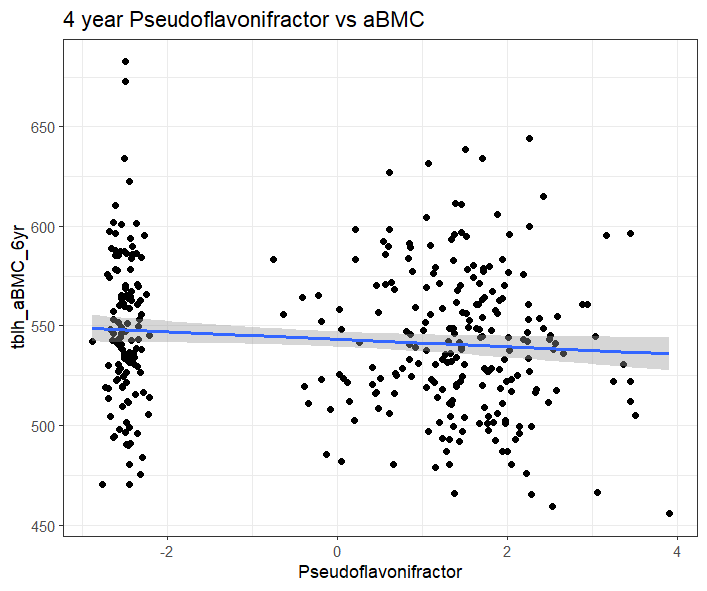
**

**
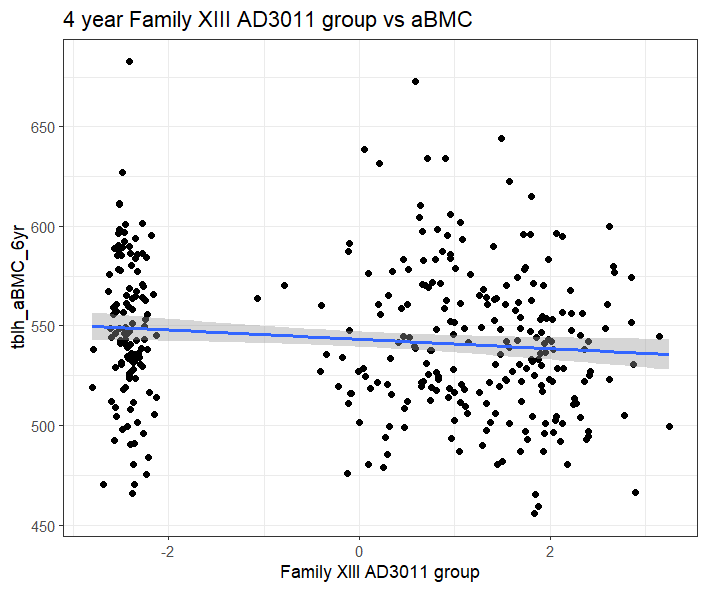
**

**Supplementary Figure 5c: Linear regression for 4-year taxa vs aBMC at 6 years**. Abundance data for taxa is centered log-ratio transformed. aBMC in grams. TBLH aBMC: Total body less head area-adjusted bone mineral content.


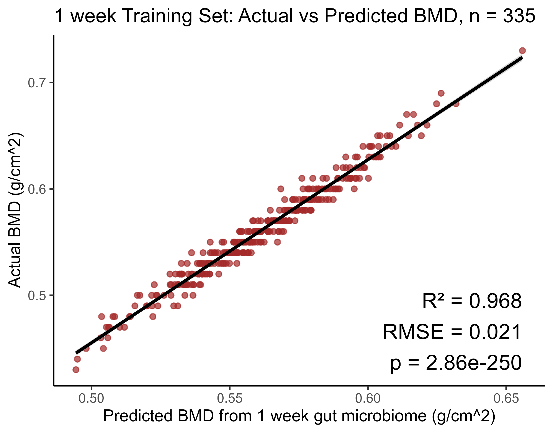

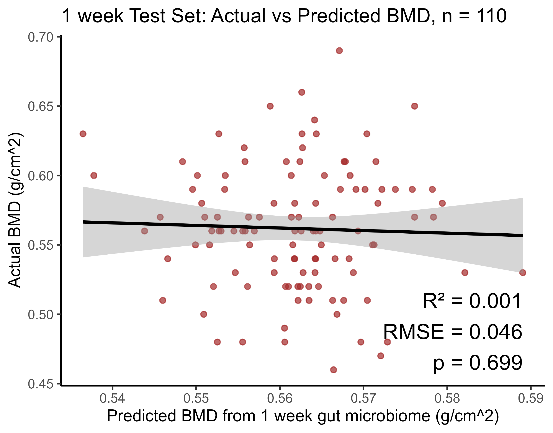

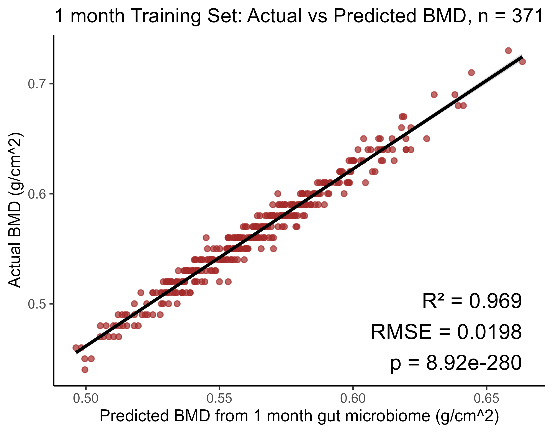

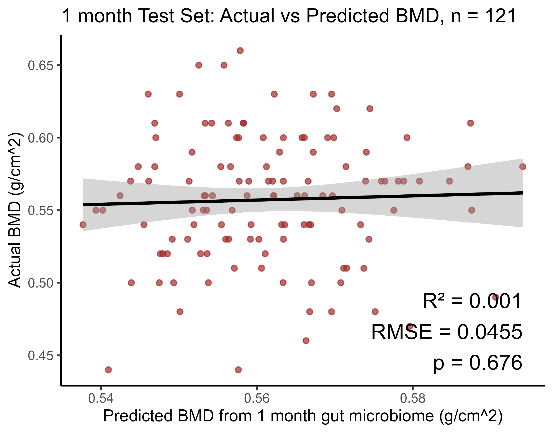

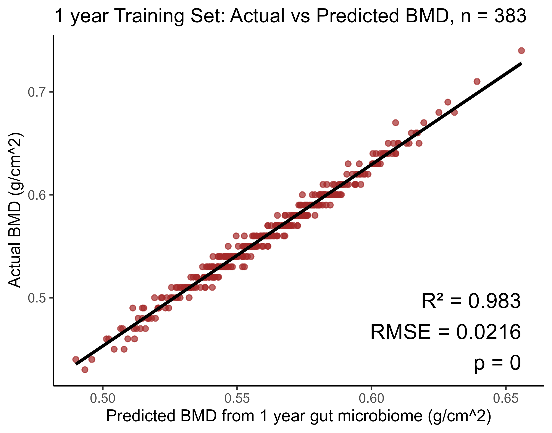

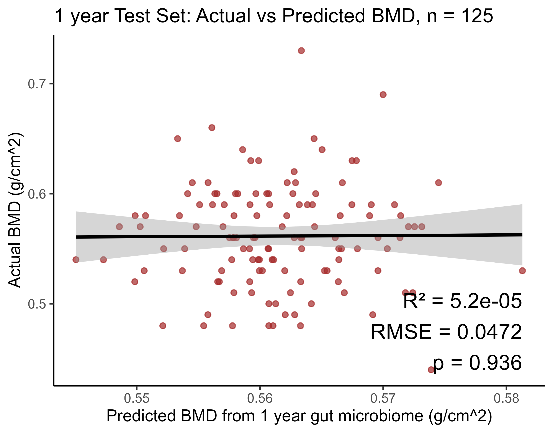

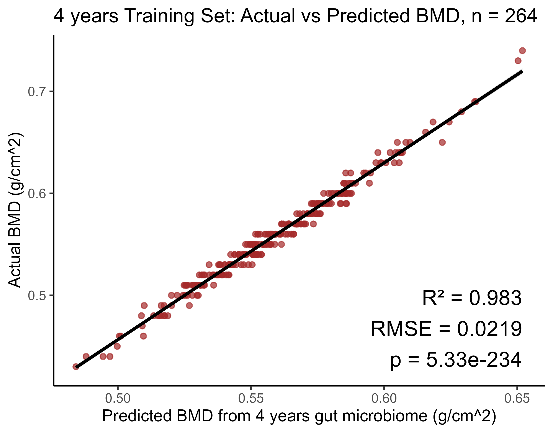

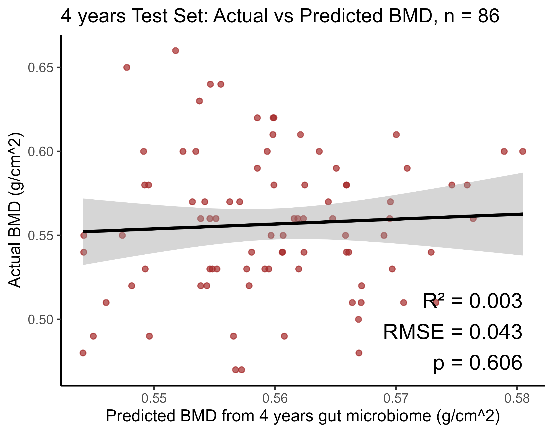

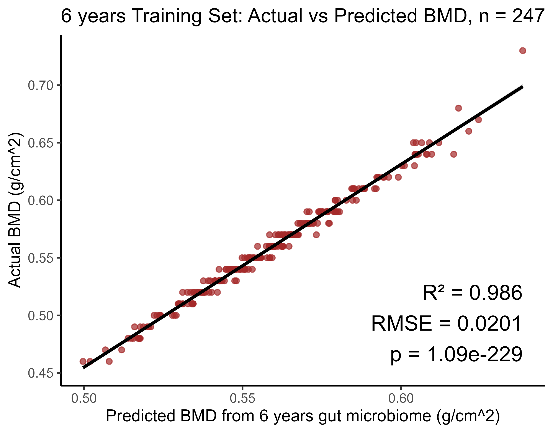

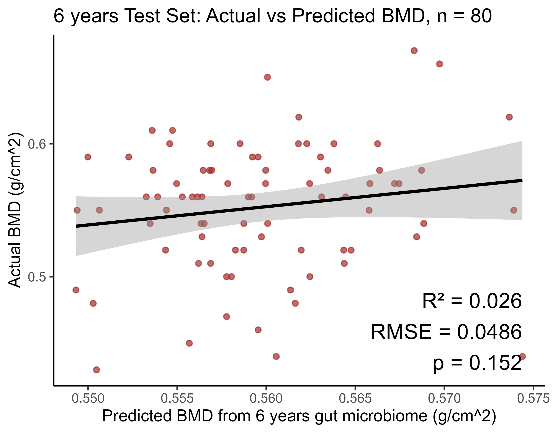


**A**

**B**

**C**

**D**

**E**

**F**

**G**

**H**

**I**

**J**

**D**

**Supplementary Figure 6a: Random Forest scatter plot of observed and predicted BMD in children at the age of 6 years based on bacterial composition at earlier ages. (A and B):** Comparison of model performance based on the training (A) and test (B) sets of 1 week data; only the training set predictions correlate with the actual values. **(C and D):** Comparison of model performance based on the training (C) and test (D) sets of 1 month data; only the training set predictions correlate with the actual values. **(E and F):** Comparison of model performance based on the training (E) and test (F) sets of 1 year data; only the training set predictions correlate with the actual values. **(G and H):** Comparison of model performance based on the training (G) and test (H) sets of 4 years data; only the training set predictions correlate with the actual values. **(I and J):** Comparison of model performance based on the training (I) and test (J) sets of 6 years data; only the training set predictions correlate with the actual values.


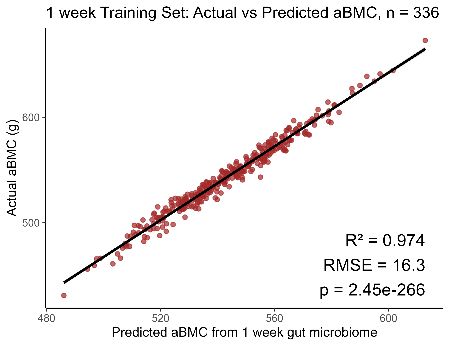

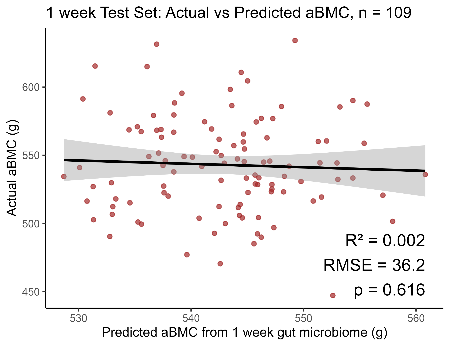

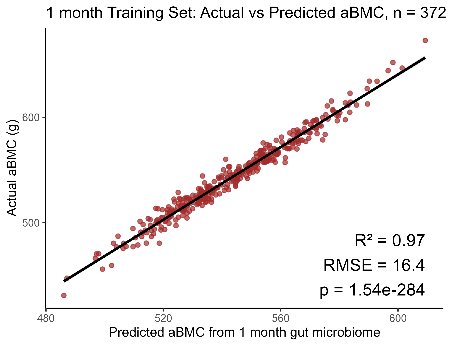

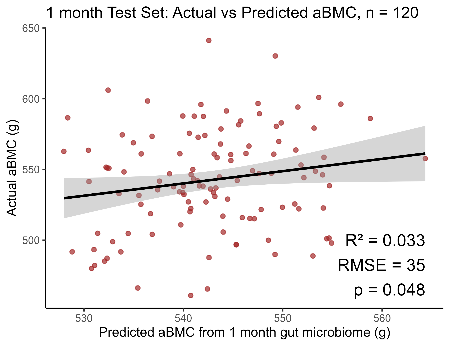

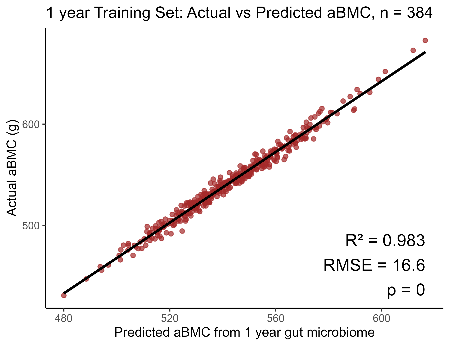

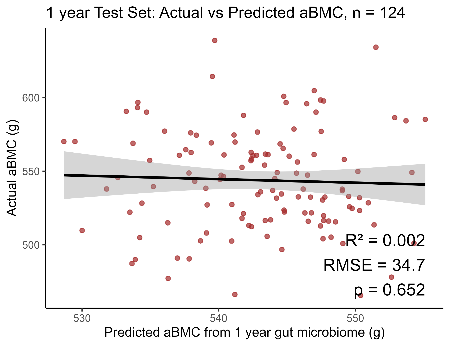

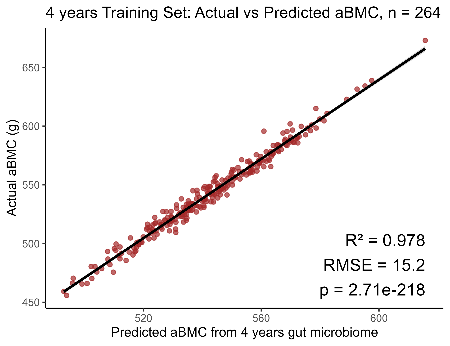

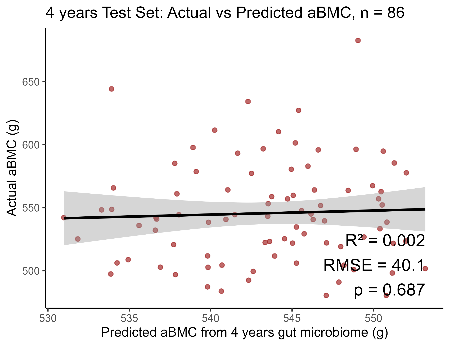

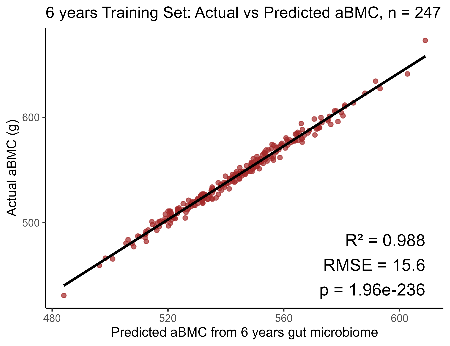

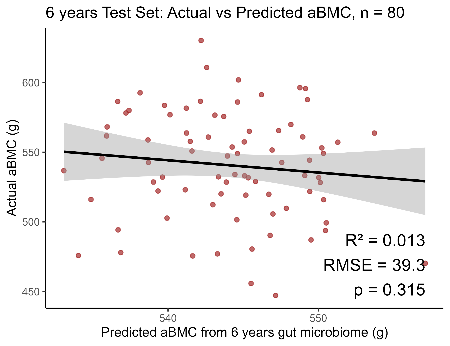


**A**

**B**

**C**

**D**

**E**

**F**

**G**

**H**

**I**

**J**

**Supplementary Figure 6b: Random Forest scatter plot of observed and predicted aBMC in children at the age of 6 years based on bacterial composition at earlier ages. (A and B):** Comparison of model performance based on the training (A) and test (B) sets of 1 week data; only the training set predictions correlate with the actual values. **(C and D):** Comparison of model performance based on the training (C) and test (D) sets of 1 month data; both training and test set predictions correlate with the actual values. **(E and F):** Comparison of model performance based on the training (E) and test (F) sets of 1 year data; only the training set predictions correlate with the actual values. **(G and H):** Comparison of model performance based on the training (G) and test (H) sets of 4 years data; only the training set predictions correlate with the actual values. **(I and J):** Comparison of model performance based on the training (I) and test (J) sets of 6 years data; both training and test set predictions correlate with the actual values.

**
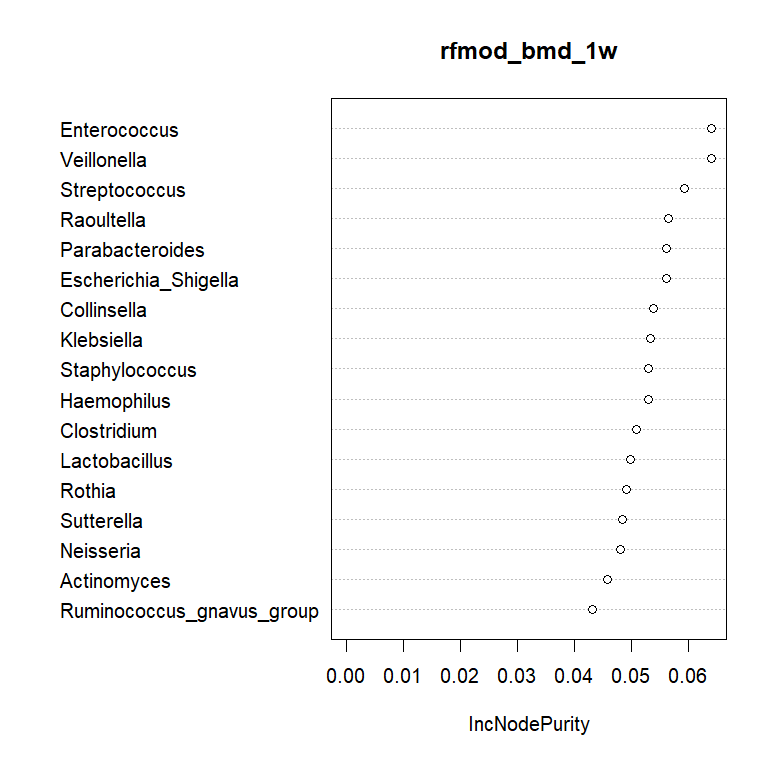

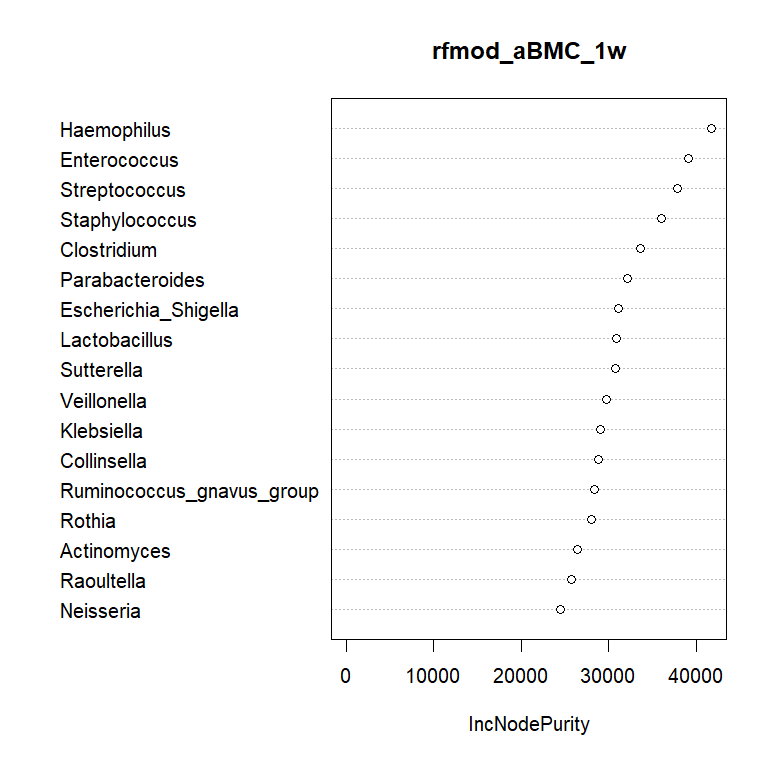
**

**Supplementary Figure 7a: Random Forest showing associations of genera with BMD and aBMC respectively at 1 week.** Ranking of important taxa with most predictive taxa at the top. Model adjusted for child’s sex, race, socio-economic status, and age, height, and bone free mass at DXA visit. IncNodePurity; represents increase in node purity which reflects the improvement in classification accuracy or predictive power when a specific variable is used to split the data in a decision tree. A higher value indicates that the variable contributes more to distinguishing between groups or outcomes by creating more uniform subgroups. Abbreviations: aBMC; area-adjusted bone mineral content, BMD; bone mineral density.

**
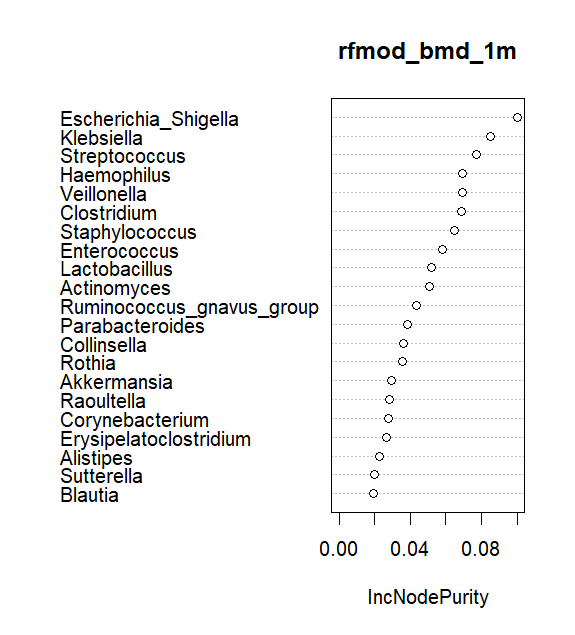

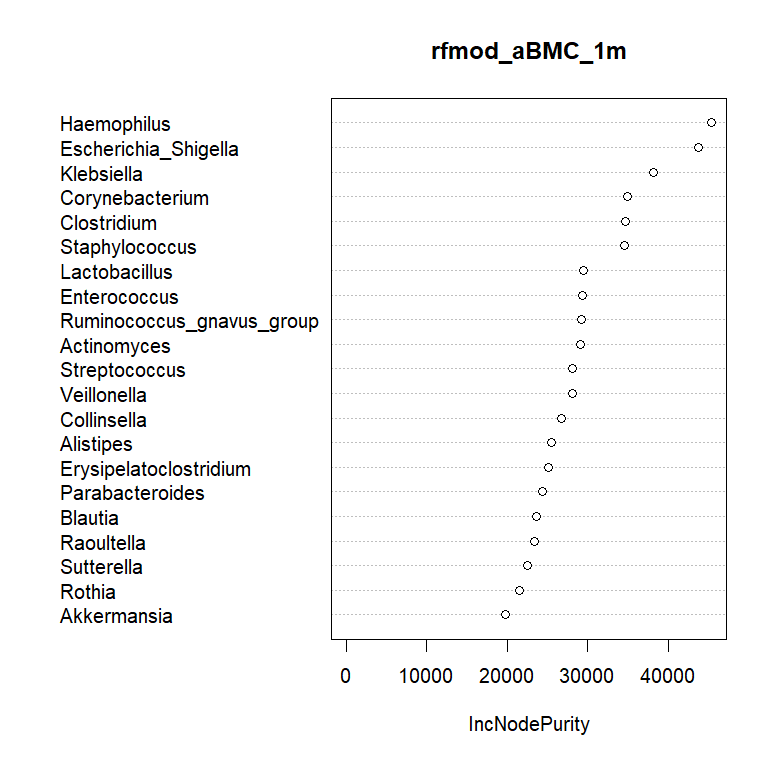
**

**Supplementary Figure 7b: Random Forest showing associations of genera with BMD and aBMC respectively at 1 month.** Ranking of important taxa with most predictive taxa at the top. Model adjusted for child’s sex, race, socio-economic status, and age, height, and bone free mass at DXA visit. IncNodePurity; represents increase in node purity which reflects the improvement in classification accuracy or predictive power when a specific variable is used to split the data in a decision tree. A higher value indicates that the variable contributes more to distinguishing between groups or outcomes by creating more uniform subgroups. Abbreviations: aBMC; area-adjusted bone mineral content, BMD; bone mineral density.


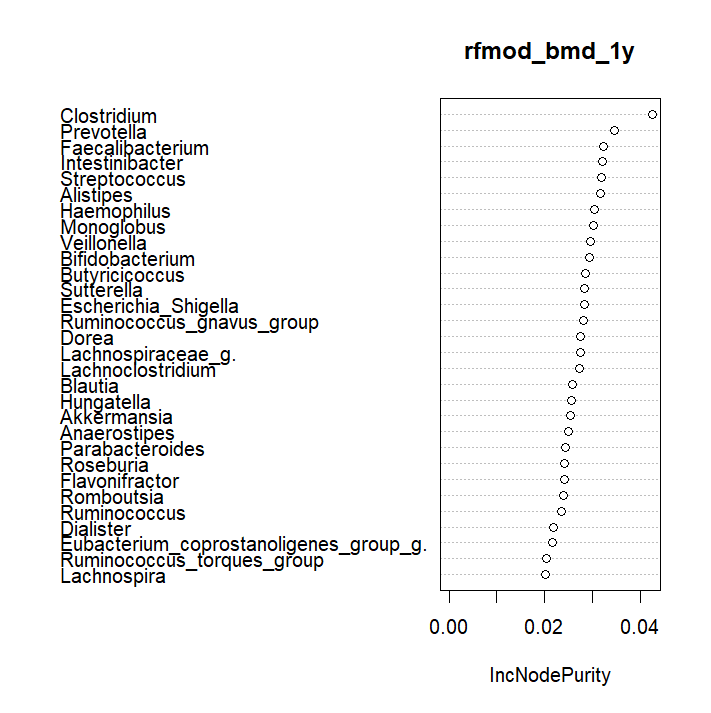
**
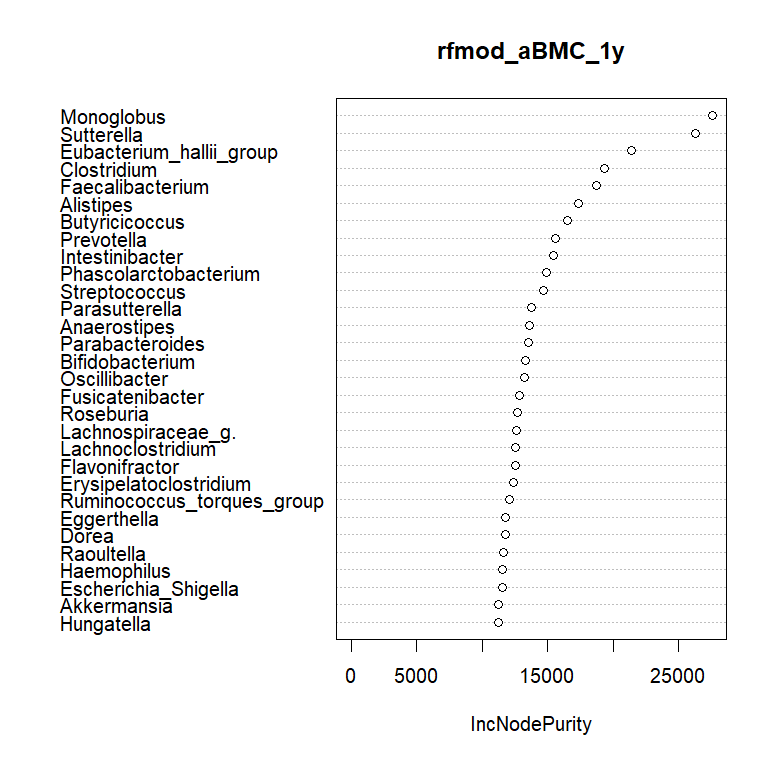
**

**Supplementary Figure 7c: Random Forest showing associations of genera with BMD and aBMC respectively at 1 year.** Ranking of important taxa with most predictive taxa at the top. Model adjusted for child’s sex, race, socio-economic status, and age, height, and bone free mass at DXA visit. IncNodePurity; represents increase in node purity which reflects the improvement in classification accuracy or predictive power when a specific variable is used to split the data in a decision tree. A higher value indicates that the variable contributes more to distinguishing between groups or outcomes by creating more uniform subgroups. Abbreviations: aBMC; area-adjusted bone mineral content, BMD; bone mineral density.

**
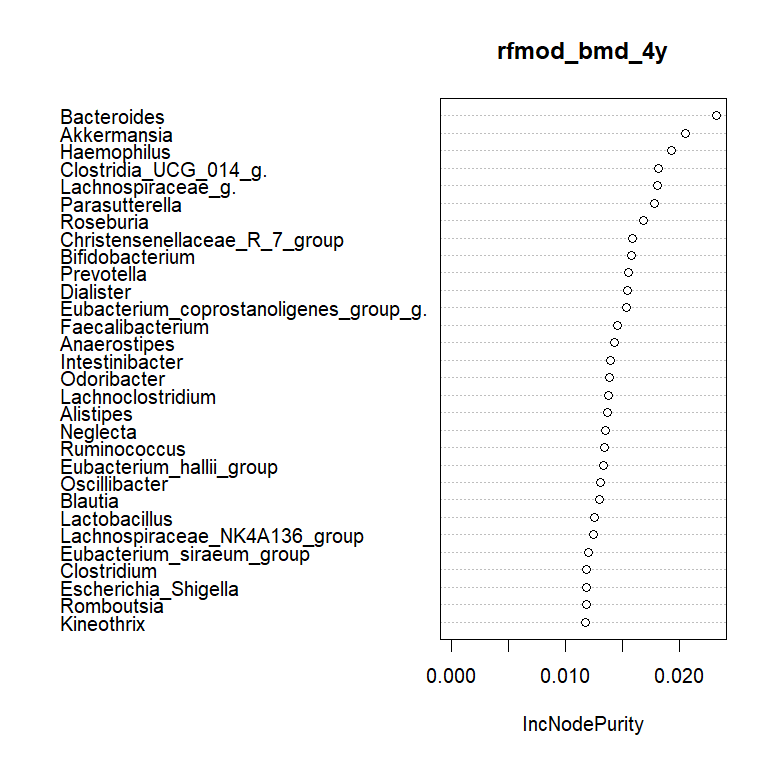

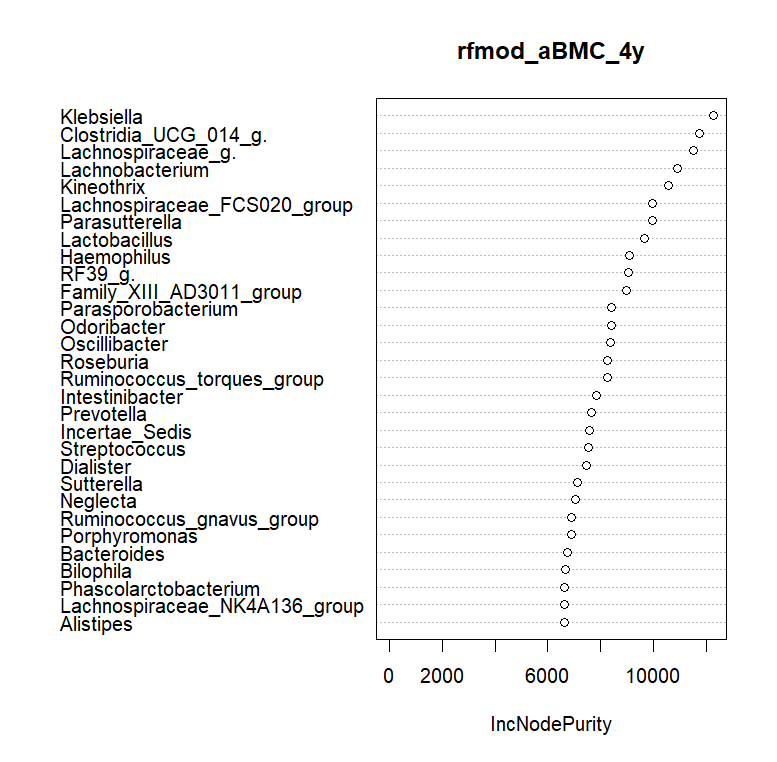
**

**Supplementary Figure 7d: Random Forest showing associations of genera with BMD and aBMC respectively at 4 years.** Ranking of important taxa with most predictive taxa at the top. Model adjusted for child’s sex, race, socio-economic status, and age, height, and bone free mass at DXA visit. IncNodePurity; represents increase in node purity which reflects the improvement in classification accuracy or predictive power when a specific variable is used to split the data in a decision tree. A higher value indicates that the variable contributes more to distinguishing between groups or outcomes by creating more uniform subgroups. Abbreviations: aBMC; area-adjusted bone mineral content, BMD; bone mineral density.

**
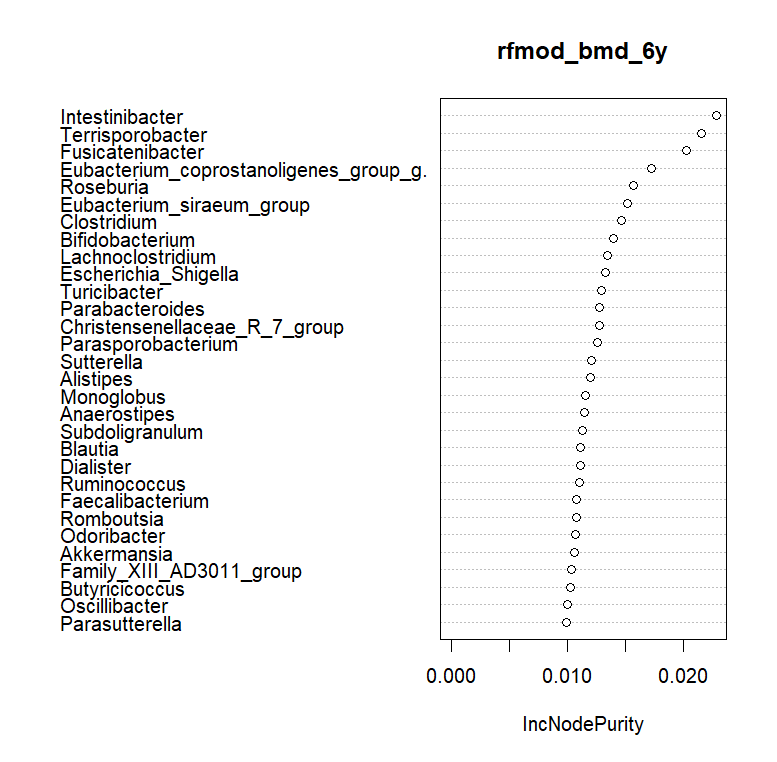

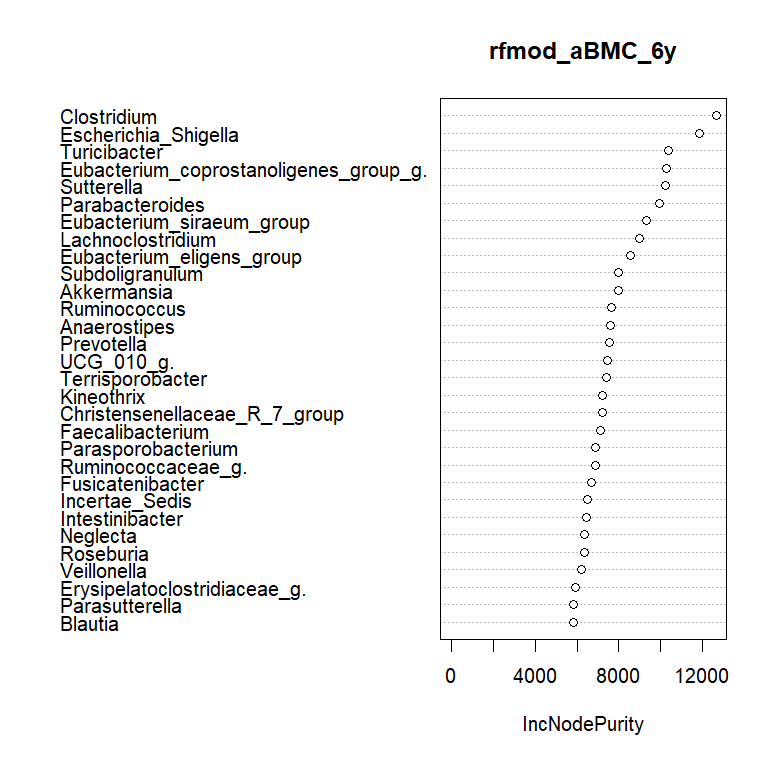
**

**Supplementary Figure 7e: Random Forest showing associations of genera with BMD and aBMC respectively at 6 years.** Ranking of important taxa with most predictive taxa at the top. Model adjusted for child’s sex, race, socio-economic status, and age, height, and bone free mass at DXA visit. IncNodePurity; represents increase in node purity which reflects the improvement in classification accuracy or predictive power when a specific variable is used to split the data in a decision tree. A higher value indicates that the variable contributes more to distinguishing between groups or outcomes by creating more uniform subgroups. Abbreviations: aBMC; area-adjusted bone mineral content, BMD; bone mineral density.


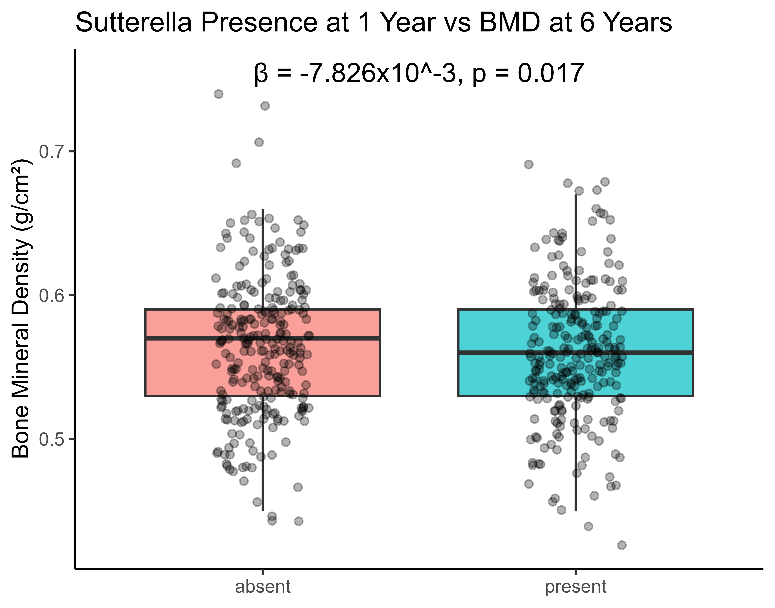

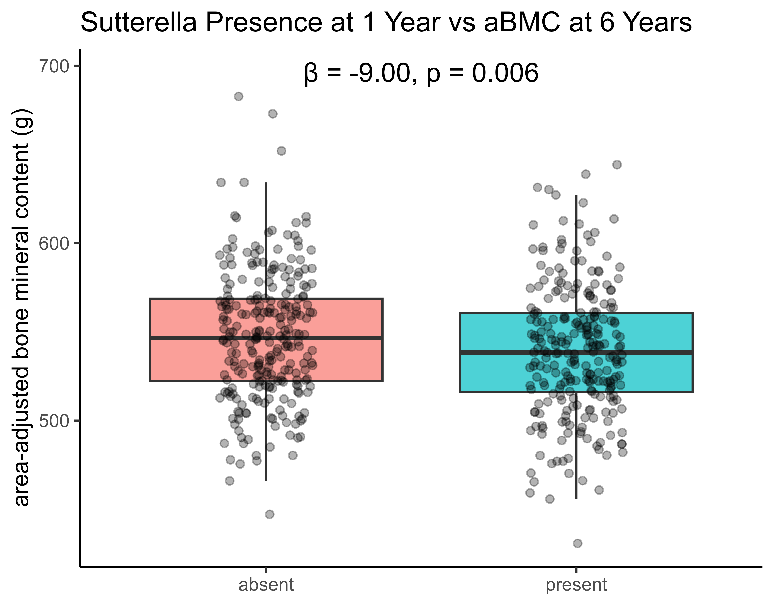


**B**

**A**

**Supplementary Figure 8: Sutterella presence/absence at 1 year and bone measures.** There is limited evidence to support the hypothesis that the association between Sutterella and bone measures are driven by its presence/absence. Between group difference was obtained by multiple linear regression with adjustment for sex, socio-economic status, race, age at 6 years, and height.


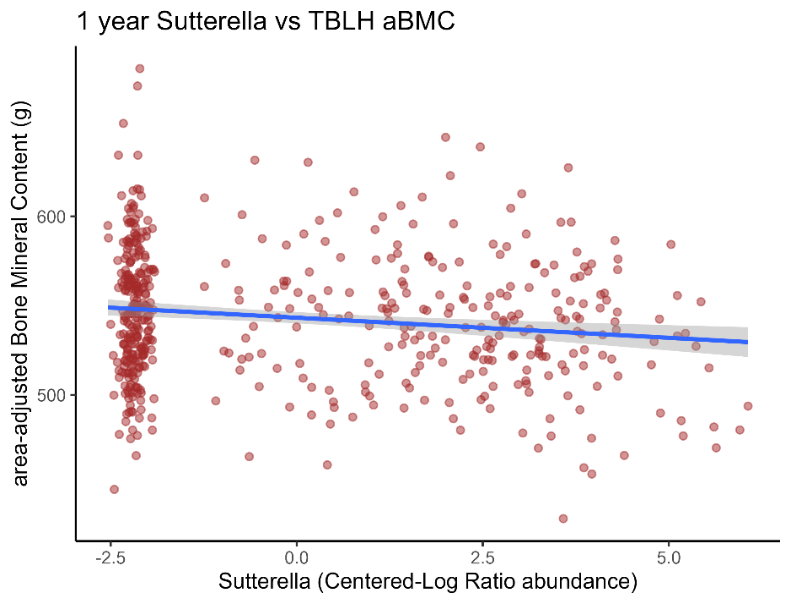

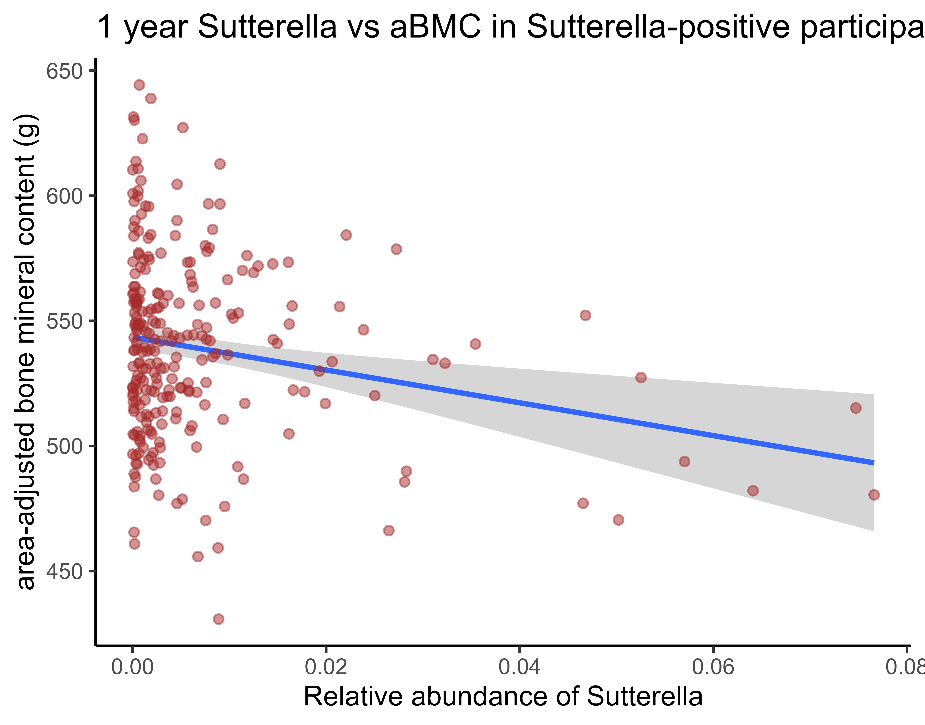


**B**

**A**

**Supplementary Figure 9: Sutterella vs aBMC at one year**. (A) Including all participants (B) Sutterella-positive participants only.
